# Anti-HIV agent azidothymidine decreases Tet(X)-mediated bacterial resistance to tigecycline in *Escherichia coli*

**DOI:** 10.1038/s42003-020-0877-5

**Published:** 2020-04-03

**Authors:** Yuan Liu, Yuqian Jia, Kangni Yang, Ruichao Li, Xia Xiao, Zhiqiang Wang

**Affiliations:** 1 College of Veterinary Medicine, Yangzhou University, Yangzhou, Jiangsu China; 2 Jiangsu Co-Innovation Center for Prevention and Control of Important Animal Infectious Diseases and Zoonoses, Yangzhou, Jiangsu China; 3 Institute of Comparative Medicine, Yangzhou University, Yangzhou, Jiangsu China

**Keywords:** Antimicrobials, Drug screening

## Abstract

Recent emergence of high-level tigecycline resistance mediated by Tet(X3/X4) in Enterobacteriaceae undoubtably constitutes a serious threat for public health worldwide. Antibiotic adjuvant strategy makes antibiotic more effective against these resistant pathogens through interfering intrinsic resistance mechanisms or enhancing antibiotic actions. Herein, we screened a collection of drugs to identify compounds that are able to restore tigecycline activity against resistant pathogens. Encouragingly, we discovered that anti-HIV agent azidothymidine dramatically potentiates tigecycline activity against clinically resistant bacteria. Meanwhile, addition of azidothymidine prevents the evolution of tigecycline resistance in *E. coli* and the naturally occurring horizontal transfer of *tet*(X4). Evidence demonstrated that azidothymidine specifically inhibits DNA synthesis and suppresses resistance enzyme activity. Moreover, in in vivo infection models by Tet(X4)-expression *E. coli*, the combination of azidothymidine and tigecycline achieved remarkable treatment benefits including increased survival and decreased bacterial burden. These findings provide an effective regimen to treat infections caused by tigecycline-resistant *Escherichia coli*.

## Abstract (web summary)

Yuan Liu et al. demonstrate that anti-HIV agent azidothymidine restores tigecycline’s activity against pathogens resistant to this antibiotic. This study suggests the combination of azidothymidine and tigecycline as an effective regimen to treat infections caused by tigecycline-resistant *Escherichia coli*.

## Introduction

The emergence of metallo-β-lactamases (MBLs)-mediated carbapenems resistance^1^ and MCR-mediated acquired colistin-resistance^2^ forces the medical workers to turn their attention on another clinically relevant antibiotic-tigecycline. Tigecycline belongs to a new group of tetracyclines called glycylcyclines, and possesses excellent oral activity and broad-spectrum antibacterial activity^3^. Importantly, different from other tetracyclines, tigecycline could circumvent the commonly tetracyclines resistance mechanisms involve efflux pump and ribosomal protection^4^. Thus, tigecycline is recognized as one of the last-resort antibiotics against super bugs, particularly for multidrug resistant Gram-negative bacteria. Previously, *tet*(X) and its variant *tet*(X2) that originally identified in Bacteroides species^5^ were found to result in tigecycline insusceptibility. But plasmid-borne *tet*(X) genes in clinical pathogens have not yet been identified before 2019. However, recent identification of plasmid-mediated high-level tigecycline resistance genes, *tet*(X3/X4)^6,7^, seriously threatens its clinical utility in treating bacterial infectious diseases. TetX and its variants are flavin-dependent (FAD) monooxygenase, which selectively hydroxylates the tigecycline substrate at C11a, resulting in the production of 11a-hydroxytigecycline and decreases its antibacterial activity^8–10^. The prevalence and rapid spread of *tet*(X) genes among pathogenic bacteria constitute a potential threat to public health. As such, there is an urgent need to explore alternative strategies against tigecycline-resistant pathogens.

Accordingly, development of novel antibiotic candidates is the most straightforward approach to address this issue^11,12^. However, recent investigations implied that this approach is not so cost-effective due to the huge time and financial investment^13^. It has been reported that developing a new medicine takes between 10 and 15 years on average and costs an average of $2.6 billion from drug discovery through FDA approval^14^. Consistently, there are only several novel antibiotics such as daptomycin^15^ were approved by FDA in the past decades. Conceivably, it is more and more difficult to mine novel lead compounds by traditional screening platform^11^. In contrast, screening potential antibiotic adjuvants from FDA-approved compounds offers a promising strategy to repurpose our existing antibiotic and overcome resistant bacteria^13,16^. Compared with other compounds, most of FDA-approved drugs have been shown to be safe under a certain dose, which acts an indispensable role in the development of new antibiotic adjuvants. For example, Aspergillomarasmine A restores meropenem activity via inhibiting MBLs activity^17^. Statins disassembles bacterial membrane microdomains and disable PBP2a oligomerization, thereby restores MRSA susceptible to penicillin^18^. In addition, pentamidine potentiates hydrophobic antibiotics activity against Gram-negative pathogens through perturbing bacterial outer membrane integrity^19^. Therefore, we reasoned that the combination of tigecycline and other FDA-approved compounds may provide a pipeline to overcome tigecycline resistance.

To that end, we evaluated the synergy effect between tigecycline and a collection of FDA-approved compounds. Encouragingly, we found that anti-HIV agent azidothymidine displays the most potent synergistic activity with tigecycline against *tet*(X4)-carrying *E. coli*. Mechanistic study demonstrated that azidothymidine is a DNA-damaging agent and potential Tet(X3/X4) inhibitor by specifically binding to its catalytic pocket. Most importantly, azidothymidine dramatically restores tigecycline activity both in vitro and in vivo. Overall, azidothymidine represents a safe and potent compound to combat the looming tigecycline resistance.

## Results

### Azidothymidine is a potent adjuvant of tigecycline

**Fig. 1:**
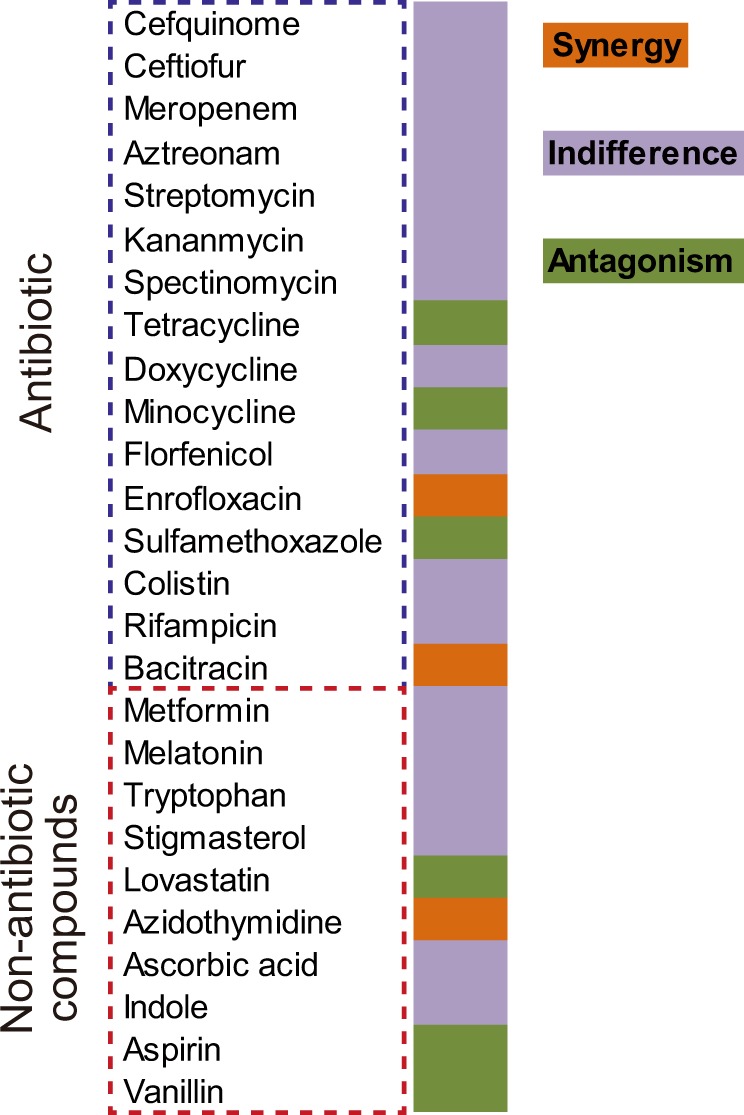
Tigecycline in combination with other representative antibiotics or nonantibiotic compounds against Tet(X4)-expressing *E. coli* B3-1. Interactions between tigecycline and compounds were divided into synergy, indifference and antagonism based on the bacterial growth inhibition rate in the presence of different combinations.

To identify potential tigecycline potentiators, we performed bacterial growth curves of *tet*(X4)-positive *E. coli* B3-1 in the presence of 26 FDA-approved compounds and/or sublethal concentration of tigecycline (8 μg mL^−1^) during 24 h culture. In particular, 26 FDA-approved compounds comprise 16 antibiotics from multiple classes, and 10 nonantibiotic compounds with different indications or usage, including antidiabetic drug (metformin), anti-insomnia drug (melatonin), essential amino acid (tryptophan), antirheumatic drug (stigmasterol), lipid-lowering drug (lovastatin), anti-HIV agent (azidothymidine), antioxidant (ascorbic acid), naturally occurring plant hormone (indole), antipyretic analgesic (aspirin), and food additive (vanillin). The *tet*(X4)-positive *E. coli* B3-1 was confirmed to be resistant to tigecycline with minimum inhibitory concentration (MIC) of 32 μg mL^−1^, which is higher than the breakpoint value (>2 µg mL^−1^) for tigecycline against Enterobacteriaceae defined by European Committee on Antimicrobial Susceptibility Testing (EUCAST). Consequently, we found that three compounds (enrofloxacin, bacitracin, and azidothymidine) with tigecycline showed above 50% growth inhibition based on the absorbance at 600 nm (Fig. 1 and Supplementary Table 1). Of particular, the combination of azidothymidine with tigecycline resulted in complete bacterial growth inhibition compared to azidothymidine or tigecycline alone, suggesting that azidothymidine may greatly potentiate tigecycline activity against *tet*(X4)-positive pathogens (Supplementary Fig. 1).

**Fig. 2:**
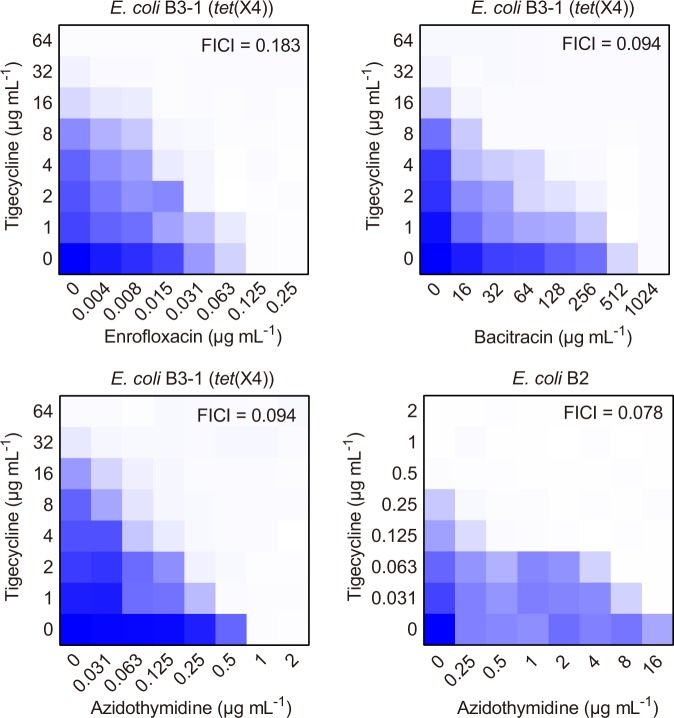
Potential tigecycline adjuvants. Synergistic activity between enrofloxacin/bacitracin/azidothymidine and tigecycline against *tet*(X4) carrying *E. coli* B3-1 or *tet*(X)-negative *E. coli* B2. Checkerboard broth microdilution assays were performed and absorbance at 600 nm after 18 h co-incubation was recorded. The blue regions represent higher cell density. Data represent the mean absorbance of two biological replicates.

To further evaluate the synergistic activity between tigecycline and three candidates, standard checkerboard microdilution in *E. coli* B3-1 was performed. As expected, the combination of tigecycline with enrofloxacin, bacitracin and azidothymidine drastically decreased the MIC values of tigecycline against *E. coli* B3-1 from 32 μg mL^−1^ to 2, 1, and 1 μg mL^−1^ (lower than the breakpoint of tigecycline by EUCAST), with the fractional inhibitory concentration indices (FIC) were determined to be 0.183, 0.094, and 0.094, respectively (Fig. 2). These results demonstrated synergistic interaction between tigecycline and three candidates (synergy was defined as an FIC index of ≤0.5). Consistent with growth inhibition test, azidothymidine displayed better potentiation activity under lower concentration than enrofloxacin and bacitracin. Thus, we next focused our insights on the potentiation potency of azidothymidine with tigecycline. We first evaluated the stability of the azidothymidine-tigecycline combination in the presence of salt ions and serum, which are two critical factors that affect the in vivo efficacy of drugs^20,21^. Consequently, we found that azidothymidine retained its activity in the presence of Na^+^, K^+^, 10% serum and 10% DMEM in medium (Table 1). However, addition of divalent cations such as Ca^2+^ and Mg^2+^ (10 mM) abolished its potentiation activity, but EDTA could augment the synergistic effect (Supplementary Fig. 2). Considering that divalent cations and EDTA are associated with membrane permeability^22^, we speculated that entrance of two drugs into bacteria contribute to their activity. Next, we assessed the potency of this combination in a panel of clinical isolates from animals and environmental samples (Supplementary Tables 2 and 3). Expected synergistic effect was observed in all test strains with FIC indices of ≤0.5 (Table 1). To investigate whether this potentiation is also applicable in Tet(X)-negative bacteria, standard ATCC sensitive bacteria and other *E. coli* isolates with MIC values of ≤1 μg mL^−1^ for tigecycline were tested. Interestingly, we found azidothymidine also showed synergism with tigecycline in these Tet(X)-negative bacteria, suggesting that azidothymidine indeed enhanced the modes of action of tigecycline (Table 1).

**Table 1:** Potentiation potency of azidothymidine with tigecycline against tigecycline resistant or sensitive clinical pathogens.

Pathogens	Source	MIC^a^ (μg mL^−1^)	MIC^b^ (μg mL^−1^)	FIC index	Potentiation (fold)^c^
TIG-resistant					
*E. coli* B3-1	Blood	32	1	0.094	32
+10 mM EDTA		8	0.063	0.039	128
+10 mM Mg^2+^		128	64	1.00	2
+10 mM Ca^2+^		64	32	1.00	2
+10 mM Na^+^		32	1	0.094	32
+10 mM K^+^		32	1	0.094	32
+10% DMEM		64	2	0.094	32
+10% serum		32	1	0.094	32
* E. coli* B8-1	Blood	32	8	0.263	4
* E. coli* B9-1		4	1	0.5	4
* E. coli* W7-1	Water	32	4	0.375	8
* E. coli* W4-1		32	0.5	0.141	16
* E. coli* W6-1		32	2	0.188	16
* E. coli* W8-1		16	0.5	0.15	32
* E. coli* S2-1	Soil	32	0.5	0.141	64
* E. coli* S3-1		32	1	0.094	32
* E. coli* S6-2		32	0.5	0.031	64
* E. coli* D5-1	Dust	16	4	0.5	4
* E. coli* T29	Trunk	32	2	0.094	16
* E. coli* T18-1		16	2	0.375	8
* E. coli* P3-1	Pork	16	1	0.188	16
* E. coli* P10-1		32	4	0.375	8
* E. coli* P12-1		16	1	0.094	16
* E. coli* F168-1	Feces	32	2	0.125	16
* E. coli* F14		32	0.5	0.031	64
* E. coli* F12-1		16	2	0.375	8
* E. brevis* S1-3	Shrimp	32	8	0.313	4
*E. fergusonnii* 1D4-6	Dust	32	2	0.094	16
*Proteus penneri* 2F1-3	Feces	32	0.25	0.023	128
*Shigella* 1F25-27		16	0.125	0.070	128
TIG-sensitive					
*S. enterica* 13076	ATCC	1	0.25	0.251	4
* E. coli* 25922	ATCC	0.25	0.063	0.5	4
* E. coli* C3	Pig	1	0.125	0.129	8
* E. coli* G6		0.5	0.125	0.266	4
* E. coli* B2		0.5	0.031	0.078	16

^a, b^MICs of tigecycline in the absence or presence of nonlethal concentrations of azidothymidine.

^c^Degree of tigecycline potentiation in the presence of nonlethal concentrations of azidothymidine.

*TIG* tigecycline, *E. brevis* *Empedobacter brevis*.

To better compare the synergistic activity of azidothymidine and tigecycline in Tet(X)-negative and positive bacteria, we determined the FIC indices in a rifampin-resistant recipient strain of *E. coli* EC600 and *E. coli* EC600 transconjugant that carried *tet*(X4)-bearing plasmid. Interestingly, we found that the potentiation activity of azidothymidine to tigecycline in transconjugant (FICI, 0.094) is higher than that in *E. coli* EC600 (FICI, 0.375) (Supplementary Table 4), indicating that the potentiation mechanisms of azidothymidine may also relate to the inhibition of the Tet(X4) enzymatic activity.

**Fig. 3:**
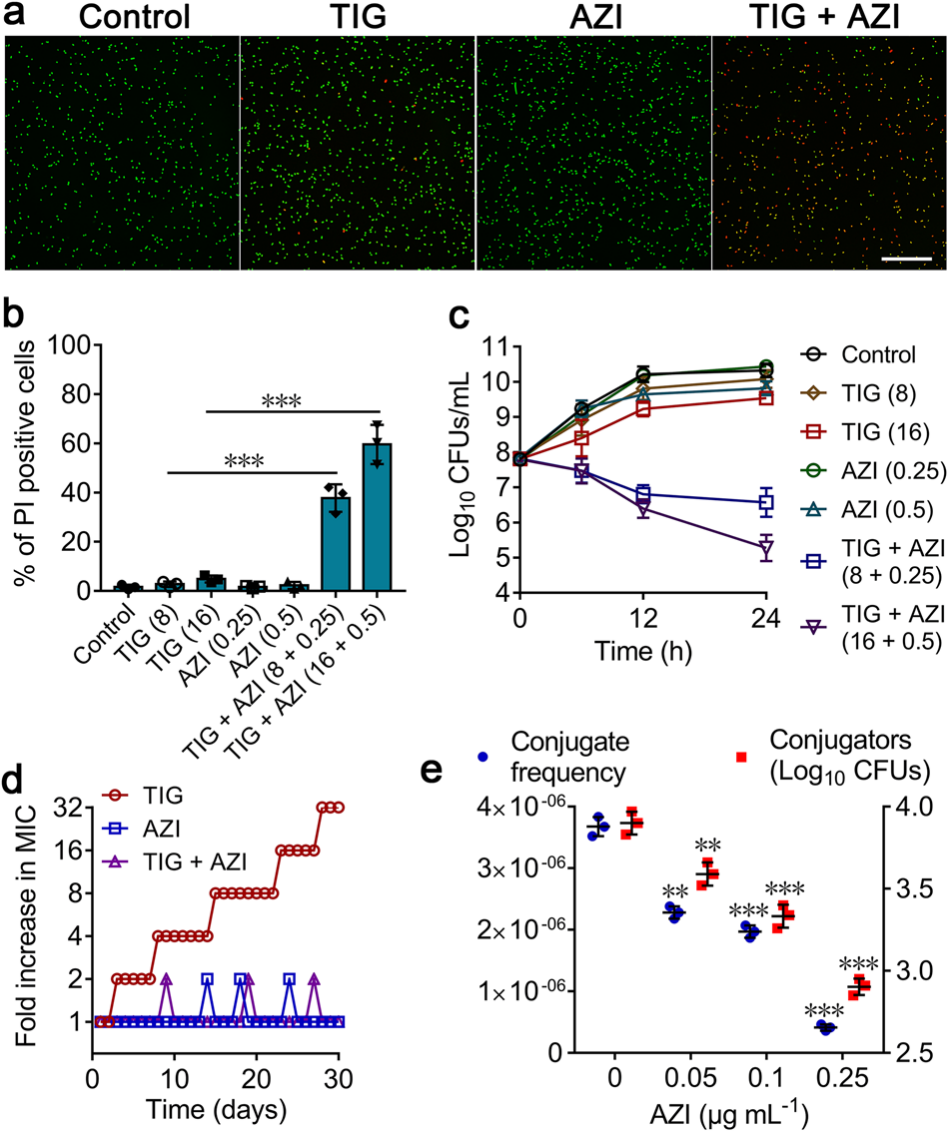
Azidothymidine potentiates tigecycline activity against resistant bacteria and prevents the evolution and spread of resistance. **a** Confocal micrographs of resuspended *E. coli* B3-1 cells after treated with PBS, tigecycline (TIG, 16 μg mL^−1^), azidothymidine (AZI, 0.5 μg mL^−1^) alone and their combination for 4 h. Viable cells were stained in green by SYTO9, whereas dead cells were in red by propidium iodide (Scale bar: 50 μm). **b** Flow cytometry analysis of propidium iodide (PI) uptake after incubation with tigecycline (TIG), or azidothymidine (AZI) or their combination at various concentrations for 4 h. All data are presented as mean ± SD and analyzed by unpaired *t* test (***P < 0.001). **c** Time-dependent killing of resistant *E. coli* B3-1 cells by the combination of tigecycline and azidothymidine. *E. coli* were grown to exponential phase and challenged with tigecycline, azidothymidine alone, and their combination during 24 h. Data are representative of three independent experiments and presented as mean ± SD. **d** Combination of tigecycline and azidothymidine prevents the evolution of resistance during 30 days serial passaging experiment. **e** Azidothymidine suppresses the horizontal transfer of *tet*(X4) from exconjugant (*E. coli* S2-1) to recipient bacteria (*E. coli* EC600). The left *y*-axis and right *y*-axis represent conjugate frequency and conjugators (Log_10_ CFUs), respectively. Experiments were performed three times, and the mean ± SD is shown. Significance compared with untreated group determined by nonparametric one-way ANOVA (**P < 0.01, ***P < 0.001). *AZI* azidothymidine, *TIG* tigecycline.

Having shown that the synergistic bacteriostatic effect of azidothymidine and tigecycline against pathogens, we set out to test whether this combination possesses synergistic bactericidal activity on resistant bacteria. LIVE/DEAD *Bac*Light viability assay were performed by a combination of two nucleic acid stains distinguishes live bacteria with an intact membrane from dead bacteria. Viable bacteria stain green with SYTO9, whereas dead bacteria appear red because of staining with propidium iodide (PI)^23^. Interestingly, we found that the combination of azidothymidine and tigecycline resulted in enhanced red fluorescence, whereas the monotreatment displayed little red fluorescence and strong green fluorescence (Fig. 3a). This result suggested that this combination could lead to bacterial cell death. To evaluate the percentage of dead bacterial cells after combination treatment, stained *E. coli* B3-1 bacterial cells by PI were incubated with various concentrations of tigecycline and/or azidothymidine and measured by flow cytometry. The percentage of PI-positive cells was greater for those treated with combination than tigecycline alone (Fig. 3b). Next, we conducted time-killing curve to assess the dynamic progress. As shown in Fig. 3c, we observed an above 3-log10 reduction of CFUs under the treatment of combination for 24 h, which confirmed the synergistic effect of this combination on tigecycline resistant pathogens.

### Azidothymidine thwarts evolution of tigecycline resistance

The emergence of resistance under long-term of exposure of drugs is an inevitable biology phenomenon^24^. Nevertheless, strategies that could delay or minimize this process were still valuable. Thus, we analyzed the ability of azidothymidine to suppress the evolution of resistance in a tigecycline-sensitive *E. coli* ATCC 25922. Sequential passaging with tigecycline alone during 30 days led to 32-fold increases in MIC, whereas the addition of azidothymidine with tigecycline showed no increase in MIC (Fig. 3d). This result indicated that azidothymidine could suppress the emergence of resistance in bacteria. Considering that plasmid-mediated horizontal transfer of resistance genes is an important cause of the prevalence and spread of drug resistance^25^, we next evaluated the effect of azidothymidine (0–1 μg mL^−1^) on conjugate frequency of *tet*(X4)-bearing plasmid. Unexpectedly, we found that transfer frequency of *tet*(X4) from exconjugants to recipients decreased in the presence of azidothymidine (0–0.25 μg mL^−1^). At higher concentration of azidothymidine (0.5 and 1 μg mL^−1^), we could not obtain any conjugators. By contrast, one quarter of MIC concentrations of other three antibiotics, including meropenem, colistin, and ciprofloxacin could not suppress the conjugate frequency of *tet*(X4)-bearing plasmid (Supplementary Fig. 3a). However, we found that sub-MIC of azidothymidine could not inhibit the conjugation of reference PR4 plasmid **(**Supplementary Fig. 3b**)**, implying that the inhibitory effect of azidothymidine on conjugation is plasmid-specific. Nevertheless, these results highlighted the potential of azidothymidine in preventing the emergence and spread of tigecycline resistance in pathogens (Fig. 3e).

### Safety evaluation of azidothymidine

Enhanced toxicity induced by the combination of two drug is a crucial concern for combination therapy^26^. To assess the effect of azidothymidine on safety of tigecycline, we performed toxicity test in red blood cells (RBCs), Chinese Hamster Ovary (CHO) cells and mice. As shown in Supplementary Fig. 4a, b, we found that tigecycline (≤128 µg/mL) showed negligible hemolytic activity to RBCs and cytotoxicity in CHO cells. Moreover, the combination of tigecycline and azidothymidine (0.5 µg/mL) displayed no any toxicity. Acute toxicity of high concentration of combination were further assessed in mice. As expected, all tested mice administrated with combination of tigecycline (100 mg kg^−1^) plus azidothymidine (10 mg kg^−1^) survived during 14 days (Supplementary Fig. 5a). Besides, this combination has no effect on growth of mice compared with tigecycline alone (Supplementary Fig. 5b). Collectively, these results illustrated that azidothymidine is a safe compound for potentiating tigecycline killing.

### Azidothymidine inhibits DNA synthesis and inactivates Tet(X)

**Fig. 4:**
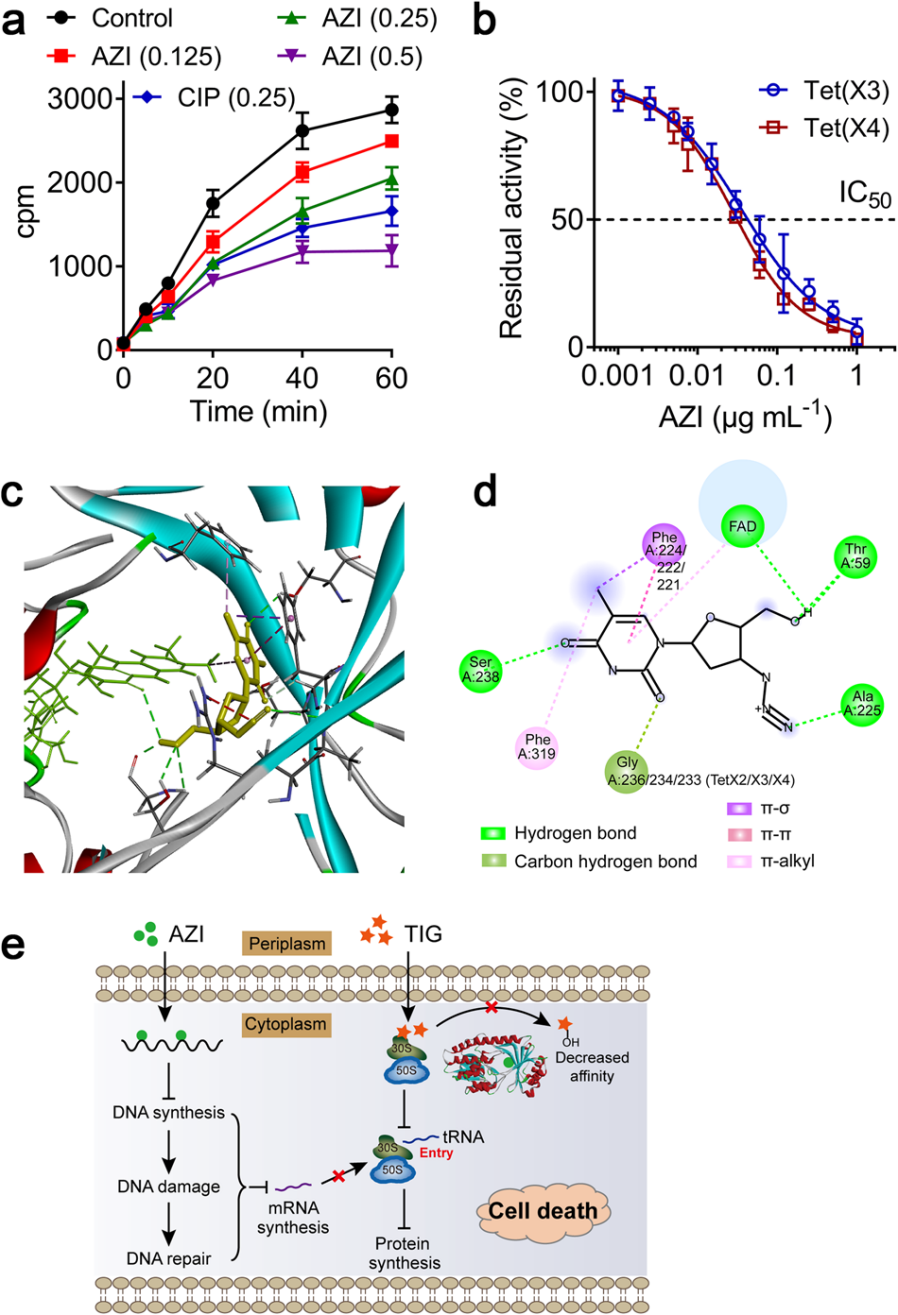
Potentiation mechanisms of azidothymidine with tigecycline. **a** Azidothymidine suppresses the DNA synthesis of *E. coli*. Incorporation of ^3^H-thymidine into DNA after treatment with sub-MICs of azidothymidine were measured. Ciprofloxacin (one-half MIC) was used as a positive control. Data are representative of three independent experiments and presented as mean ± SD. **b** Azidothymidine inhibits *tet*(X3/X4) activity purified from *E. coli* B3-1 in a dose-dependent manner (IC_50_, 0.0358 and 0.0284 μg mL^−1^). Data are representative of three independent experiments and presented as mean ± SD. **c** Molecular docking analysis of the complexes of Tet(X2/3/4) with FAD and azidothymidine. Structure of complexes of Tet(X2/3/4) with FAD (green) and azidothymidine (yellow). **d** Interactions between azidothymidine and the residues of the binding sites in Tet(X2/X3/X4) are shown using a two-dimensional diagram. **e** Scheme of azidothymidine potentiates tigecycline activity. Azidothymidine enhances tigecycline activity through two pathways: (1) Azidothymidine inhibits DNA synthesis and leads to DNA damage and SOS response, which subsequently affects synthesis of specific mRNA. This action displayed a complementary mechanism with tigecycline in inhibiting protein synthesis. (2) Azidothymidine could localize to Tet(X) catalytic pocket and inhibit its enzymatic activity, then restore tigecycline activity against resistant bacteria.

Considering that azidothymidine potentiates tigecycline activity in both resistant and sensitive bacteria, we reasoned that azidothymidine may performed a complementary mode of action with tigecycline. It has been proved that azidothymidine is nucleoside reverse transcriptase inhibitor and widely used to treat HIV infection^27^. Inspired by this, we firstly investigate the effect of azidothymidine on DNA synthesis. As shown in Fig. 4a, we found that bacterial cells exposed to sub-MICs of azidothymidine stopped incorporating radioactive DNA in a dose-dependent manner, indicating that azidothymidine substantially inhibited the rate of DNA synthesis. mRNA is generated from a strand of DNA as a template and employed as template of protein synthesis. Thus, we speculated that the induced-DNA damage may inhibit the generation of mRNA. As expected, the transcription of metabolism-related genes including *sdhC* (succinate dehydrogenase complex subunit C) and *mdh* (malate dehydrogenase), and resistance gene *tet*(X4) in *E. coli* showed dose-dependent inhibition by azidothymidine (Supplementary Fig. 6). Meanwhile, an induction of SOS response and DNA repair due to the upregulation of *recA* and *lexA* expression was observed (Supplementary Fig. 7). Thus, we concluded that azidothymidine and tigecycline acts synergy by inhibiting different stage of protein synthesis, including generation of mRNA and interaction of tRNA with ribosome. Consistently, another DNA-damaging agent enrofloxacin also showed synergistic activity with tigecycline. However, we did not observe similar synergy effect between danofloxacin^28^ (an FDA-approved animal fluoroquinolones) and tigecycline, suggesting the interaction of tigecycline and DNA-damaging compounds is drug-specific.

Our study has showed that the addition of EDTA enhanced the synergy effect of azidothymidine and tigecycline, whereas Mg^2+^ abolished its activity. Thus, we reasoned whether azidothymidine also permeabilizes the membrane permeability. To test this, we evaluated membrane permeability related biochemical parameters, including outer membrane, membrane potential and ROS generation. However, addition of azidothymidine has no significant effect on membrane associated factors, indicating that the potentiation activity of azidothymidine is not dependent on membrane disruption (Supplementary Fig. 8). This result is consistent with the MIC analysis that EDTA and Mg^2+^ have no effect on antibacterial activity of azidothymidine alone (Supplementary Table 5). However, we observed decreased tigecycline activity (four-fold MIC increases) in the presence of Mg^2+^ (10 mM) (Supplementary Table 5). Taken these results, we concluded that the promotion of EDTA and inhibition effect of Mg^2+^ on their synergistic activity is associated with change of tigecycline activity, but does not correlate with azidothymidine. Nevertheless, this phenomenon implied that the membrane-damaging agents could further strengthen the synergy effect between azidothymidine and tigecycline against pathogens.

Given that *tet*(X4)-expressing transconjugants displayed enhanced susceptibility to this combination than the negative exconjugant bacteria, we reasoned that azidothymidine may also affect the activity of resistance enzymes. To test this, we constructed *tet*(X4)-bearing expressing vector in *E. coli* BL21 and obtained the purified Tet(X3/X4) proteins, and performed enzyme activity inhibition assay in the presence of increasing concentrations of azidothymidine. Excitingly, we found that the absorbance of tigecycline at 400 nm elevated as azidothymidine increases despites of the presence of Tet(X3/X4). Consequently, we observed a dose-dependent inhibition on the enzymatic activities of Tet(X3/X4) with IC_50_ of 0.0358 and 0.0284 μg mL^−1^ (0.134 and 0.106 μM), respectively (Fig. 4b).

To better understand how azidothymidine inhibits the enzymatic activity of Tet(X), we performed in silico docking analysis between Tet(X) as receptor and azidothymidine as the ligand. Interestingly, we found that azidothymidine could localize to Tet(X) catalytic pocket. Specifically, azidothymidine could form hydrogen bonds with amino acid residues (Thr59, Ala225, Gly236, and Ser238), π–σ or π–π interaction with Phe224. Among, Phe224 and Gly236 are present in substrate-binding pocket of Tet(X) relate proteins that are crucial for inactivation of tigecycline^9^. Azidothymidine showed a high affinity with three Tet(X2/X3/X4) proteins, with −7.8, −8.8, and −7.1 kcal mol^−1^ binding energies, respectively (Fig. 4c, d). These data demonstrated that azidothymidine potentiates tigecycline activity through damaging bacterial DNA synthesis and inhibiting FAD-monooxygenase Tet(X3/X4) activity (Fig. 4e).

### Azidothymidine reverses tigecycline resistance in vivo

**Fig. 5:**
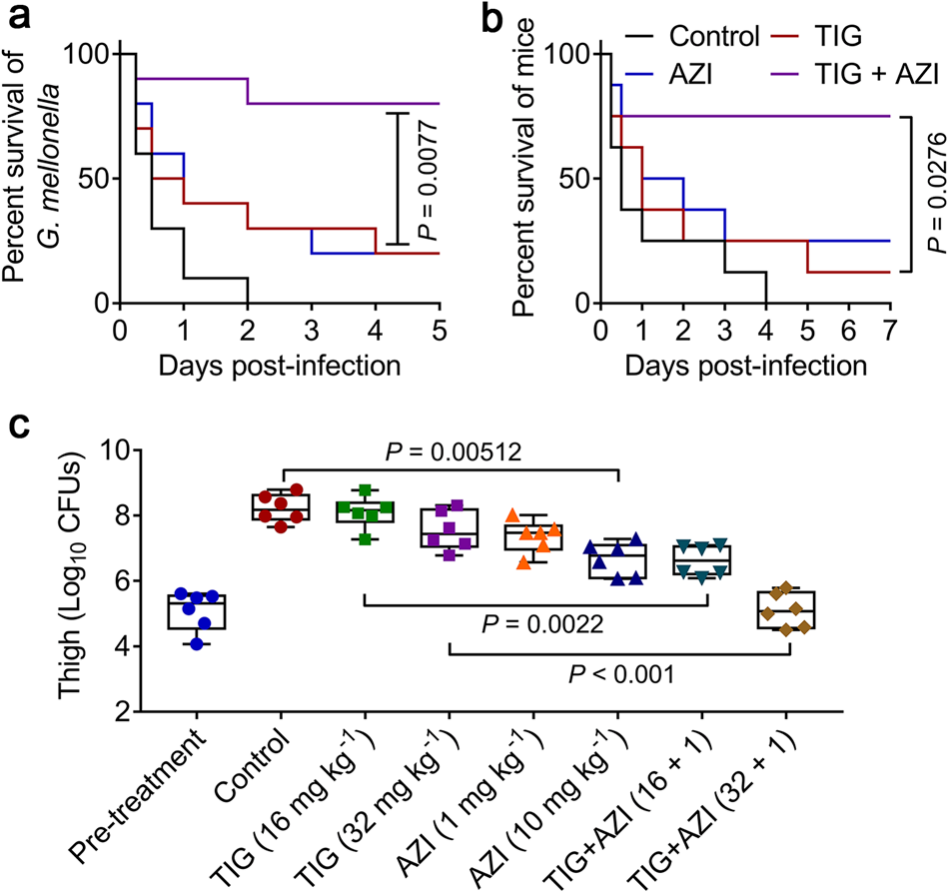
Azidothymidine rescues tigecycline activity in vivo. **a**, **b** In vivo synergy between tigecycline and azidothymidine in a *G. mellonella* larvae (**a**) and mouse peritonitis model (**b**). *G. mellonella* larvae (*n* = 10 per group) or CD-1 female mice (*n* = 8 per group) were infected with a lethal dose of *E. coli* B3-1. After 2 hours post infection, mice were treated with a single dose of PBS, tigecycline (32 mg kg^−1^), azidothymidine (1 mg kg^−1^) or combination of tigecycline (32 mg kg^−1^) plus azidothymidine (1 mg kg^−1^) by intraperitoneal injection. Survival of *G. mellonella* larvae and mice were recorded at 5- or 7-days post infection, respectively. *P* values were determined by log-rank (Mantel–Cox) test. **c** Bacterial burden of right thigh muscle in a neutropenic mouse thigh infection model by a nonlethal dose of *E. coli* B3-1 significantly decreased after a single intraperitoneal dose of combination treatment. *P* values were determined by Mann–Whitney *U* test.

Given that azidothymidine effectively restored tigecycline activity in vitro, we next sought to investigate the potential of azidothymidine to rescue tigecycline activity in vivo. We first tested the pharmacokinetic characters of two drugs in mice after a single i.p. injection by optimized liquid chromatography mass spectrometry (LC–MS/MS) analysis. Interestingly, we found that tigecycline and azidothymidine displayed similar concentration-time curves and comparable pharmacokinetic parameters, such as Tmax (time to maximal drug concentration) and Vz_F (distribution volume of drug) (Supplementary Fig. 9). This result implied that this combination may maximize their synergistic antibacterial activity in vivo. To investigate the therapeutic potential of this combination, *Galleria mellonella* and mice infection models infected by Tet(X4)-expressing *E. coli* were constructed and employed. In *Galleria mellonella* larvae model, we found that the combination therapy obtained significant survival benefit compared with tigecycline alone (*P* = 0.0077) (Fig. 5a). Consistently, the tigecycline and azidothymidine monotherapy resulted in 12.5% and 25% survival, whereas the combination therapy obtained 75% survival in mice peritonitis model (Fig. 5b). This combination therapy was further confirmed in a neutropenic mouse thigh infection model. Consequently, the combination therapy (16 + 1 or 32 + 1 mg kg^−1^) demonstrated potent bactericidal activity (approximate 2log reduction of CFU) than tigecycline alone (*P* = 0.0022 or *P* < 0.001, respectively) (Fig. 5c). Interestingly, we found that higher dose of azidothymidine monotherapy (10 mg kg^−1^) also significantly reduced bacterial burden in the mice thigh (*P* = 0.00512). Taken together, these in vivo efficacy data underlined the huge potential of azidothymidine to rescue tigecycline for treating several types of infection caused by Tet(X3/X4)-expressing tigecycline-resistant pathogens.

## Discussion

Antibiotic resistance in bacteria has been recognized as one of the most serious global threats to human health^29,30^. It is estimated that the antibiotic resistant bacteria would kill 10 million lives a year and lead to 100 trillion USD of economic loss by 2050^31^. Compared with Gram-positive pathogens, Gram-negative pathogens are more difficult-to-treat due to their highly impermeable outer membrane, which serves as a barrier to many otherwise effective antibiotics^32^. The rapid prevalence of carbapenem-resistant Enterobacteriaceae compromised the efficacy of carbapenems and treatment options are mainly dependent on colistin and tigecycline^33,34^. However, the emergence of plasmid-mediated colistin-resistance genes (*mcr*) in 2016 diminished the clinical potential of colistin^2^. Thus, tigecycline was recognized as the only last-resort of defense against multidrug resistant pathogens. Unfortunately, the identification of acquired high-level of tigecycline resistance in clinical isolates in 2019 undoubtably trigger the alarm for our medical system^6,7^. There is urgent need to explore timely and effective strategies to cope with this challenge. Drug combination offer a productive strategy to address this challenging problem^26,35^. In this study, we discovered that three compounds including enrofloxacin, bacitracin and non-antibacterial agents azidothymidine, effectively potentiated in vitro activity of tigecycline against resistant pathogens. Of particular, azidothymidine showed the greatest activity at a lower concentration and thereby was performed for our further assessment. Consistently, a previous study has also demonstrated some preliminary evidence that the synergistic activity between azidothymidine and tigecycline in the carbapenem-resistant Enterobacteriaceae^36^, but the underlying modes of action and potential synergistic activity in *tet*(X)-positive pathogens are still unknown.

Accordingly, azidothymidine (3′-azido-3′-deoxythymidine) is a thymidine analog and a potent inhibitor of retrovirus replication^37^. In clinic, azidothymidine has been applied for treatment of human immunodeficiency virus (HIV) infection and acquired immunodeficiency syndrome (AIDS) over 30 years^38^. In addition to antivirus activity, several studies have demonstrated the in vitro antimicrobial properties of azidothymidine against Enterobacteriaceae including *K. pneumoniae*^39,40^. Meanwhile, azidothymidine has shown in vitro synergistic activity with colistin^41^ or other small antimetabolite molecules such as floxuridine^42^ against resistant Enterobacteriaceae clinical isolates. It has been indicated that azidothymidine acts as a DNA chain terminator owing to the substitution of 3-hydroxyl group in thymidine by an azide group, which thereby prevents the incorporation of DNA polymerases with the following nucleotide^43^. Consistently, we also observed an obvious inhibition effect on bacterial DNA synthesis by azidothymidine. It has been found that bacteria, like eukaryotic cells, possess mechanisms of programmed cell death (PCD) under specific stimulation, such as structurally nanoengineered antimicrobial peptide polymers (SNAPPs)^44^. Two major PCD pathways including *recA* and *lexA* genes mediated apoptotic-like death (ALD) pathway and *mazEF* pathway have been reported in bacteria^45,46^. Our study revealed that the inhibition of azidothymidine on DNA synthesis would further trigger bacterial DNA repair and induce ALD through upregulation of *rec*A and *lex*A, which may subsequently lead to cell lysis^47^. These evidences provide a mechanistic explanation for its antibacterial activity.

In addition, inhibition of DNA synthesis directly affects the generation of mRNA, which acts as a template in protein synthesis. In contrast, tigecycline specifically binds to the 30S ribosomal subunit of bacteria and thereby blocks the interaction of aminoacyl-tRNA with the A site of the ribosome. It suggests that these two drugs displayed complementary antibacterial mechanisms in inhibiting protein synthesis. This also provides an interpretation on their broad-spectrum synergistic activity against both tigecycline sensitive and resistant pathogens. Consistently, another DNA-damaging agent such as enrofloxacin was also found to potentiate tigecycline activity. However, another fluoroquinolone antibiotic named danofloxacin displayed no synergy effect with tigecycline. Chemical structure differences of piperazine rings at position 7 may account for this confusing result^48^, but the detailed reasons are still unclear. In addition, it is interesting to explore the underlying mechanism by which divalent cations-dependent bacitracin potentiates tigecycline activity. Most importantly, we found that azidothymidine is also a potent inhibitor of newly reported tigecycline resistance enzymes Tet(X3/X4) through enzyme inhibition assay, which explain its better potentiation activity in resistant *E. coli*. A preliminary molecular docking analysis showed that azidothymidine could localize to the catalytic pocket of Tet(X3/X4), thereby block the binding of substrate with enzymes and deprive its biological activity. However, more investigations such as crystallographic analysis are still needed to elucidate the accurate binging sites between azidothymidine and Tet(X3/X4).

Our acute toxicity experiments demonstrated that the addition of azidothymidine (10 mg kg^−1^) has no effect on survival and body weight of mice during 14 days. Meanwhile, our study showed that the combination of low dose of azidothymidine (1 mg kg^−1^) and tigecycline could effectively treat resistant bacteria in three animal infection models. These results confirmed the safety and effectiveness of azidothymidine at therapeutic dose. Certainly, the development of targeted drug delivery based on biomaterials such as nanoparticle^49^ and liposomes^50^ may further improve its efficacy and reduce side effects.

In conclusion, we demonstrated that anti-HIV agent-azidothymidine is a potent tigecycline adjuvant in the fight against tigecycline-resistant *E. coli* both in vitro and in vivo. Specifically, azidothymidine potentiates tigecycline activity through inhibiting bacterial DNA synthesis and inactivating tigecycline resistance enzymes Tet(X3/X4). Further studies are required to verify the efficacy of this combination treatment in other notorious Gram-negative pathogens such as *Acinetobacter baumannii*. Nevertheless, the discovery of promising tigecycline adjuvant extends our pipelines to address the emerging threat of tigecycline-resistant *E. coli* infections.

## Methods

### Bacterial and chemical reagents

All strains used in this study are listed in Supplementary Table 2. Unless otherwise noted, strains were grown in Mueller–Hinton Broth (MHB, Qingdao Hope Bio-technology, China) or on MH agar plates at 37 °C. CHO cells were grown in dulbecco’s modified Eagle’s medium (DMEM, Gibco) supplemented with 10% fetal bovine serum (FBS, Invitrogen), 1% (w/v) penicillin–streptomycin and 1% (w/v) sodium pyruvate (Sigma-Aldrich, Dorset, UK). Antibiotics were obtained from China Institute of Veterinary Drug Control and other chemical reagents were purchased from Aladdin (Shanghai, China) or TCI (Shanghai, China).

### Antibacterial test

MICs of all compounds were determined by the standard broth microdilution method, based on the CLSI 2016 guideline^51^. Briefly, drugs were twofold diluted with MHB, and then mixed with an equal volume of bacterial suspensions (1.5 × 10^6^ colony-forming units (CFUs) mL^−1^) in a clear 96-well U-shape microliter plate (Corning, NY, USA). After 18 h incubation at 37 °C, the MIC values were determined as the lowest concentration of compounds at which no bacterial growth was detected.

### Primary screening with FDA-approved compounds

Antibiotic and non-antibacterial compounds (one quarter of MICs) were added to MHB medium containing 8 µg/mL tigecycline in 96-well plates. Then, 100 µL bacterial suspension (1.5 × 10^6^ CFUs) of *E. coli* B3-1 were added to the plates and co-incubated at 37 °C for 24 h. Bacterial growth in the presence of tigecycline, compounds alone or combination was monitored by measuring the absorbance at 600 nm. MHB medium containing tigecycline (8 µg/mL) with or without bacteria were served as positive or negative controls, respectively. Bacterial growth inhibition rate (%) was calculated as (ODpositive control − ODsample)/(ODpositive control − ODnegative control) × 100%. Synergy, indifference, and antagonism were defined as the inhibition rate of ≥50%, 0–50%, and <0%, respectively^52^.

### Checkboard assays

Bacteria were grown overnight in MHB and diluted 1/5000 into fresh MHB media. Synergistic activities of tigecycline and compounds were determined by checkerboard assays with eight twofold serially dilution of drugs in final volumes of 200 µL, to create an 8 × 8 matrix. After 18 h co-incubation with bacterial suspension (1.5 × 10^6^ CFUs), the absorbance at 600 nm were measured by Microplate reader. At least two biological replicates were performed for each combination and the means were used for FIC calculation according to the formula as follows^53,54^:

$${\mathrm{FIC}}\;{\mathrm{index = MIC}}_{{\mathrm{ab}}}{\mathrm{/MIC}}_{\mathrm{a}} + {\mathrm{MIC}}_{{\mathrm{ba}}}{\mathrm{/MIC}}_{\mathrm{b}} = {\mathrm{FIC}}_{\mathrm{a}} + {\mathrm{FIC}}_{\mathrm{b}}.$$

MIC_a/b_ is the MIC of compound A/B alone; MIC_ab_ is the MIC of compound A in combination with compound B; MIC_ba_ is the MIC of compound B in combination with compound A; FIC_a/b_ is the FIC of compound A/B. Synergy is defined as an FIC index of ≤0.5.

For assessing the effect of serum, DMEM, EDTA, and metal ions on the synergistic activity between tigecycline and azidothymidine, MHB containing 10% serum, 10% DMEM, 10 mM EDTA, Na^+^, K^+^, Mg^2+^, and Ca^2+^ were used in checkboard assays.

### Time-dependent killing

Tigecycline-resistant *E. coli* B3-1 was grown overnight and diluted 1/1000 in MHB, and incubated at 37 °C for 6 h. Bacteria was then challenged with phosphate-buffered saline (PBS) (control), tigecycline (16 μg mL^−1^) and azidothymidine (0.5 μg mL^−1^) monotherapy or combination therapy for 24 h in culture tubes. At intervals, 100 μL aliquots were removed, centrifuged at 10,000*g* for 1 min and resuspended into 100 μL sterile PBS. Subsequently, ten-fold serially diluted suspensions were plated on MHA plates and incubated overnight at 37 °C. Bacterial colonies were counted and the primary CFU/mL was calculated. All experiments were performed with at least three biological replicates.

### Safety assessment

Effect of azidothymidine on the hemolytic activity of tigecycline to RBCs was evaluated as follows^55,56^. Briefly, 8% sheep blood cells that prepared from fresh sterile defibrinated sheep blood, was equal volume treated with the combination of tigecycline (16–128 μg mL^−1^) with azidothymidine (0.5 μg mL^−1^) at 37 °C for 1 h. PBS (0.01 M, pH = 7.4) in the presence or absence of 0.2% Triton X-100 was used as a positive control and negative control, respectively. The absorption of released hemoglobin was measured at 576 nm by Infinite M200 Microplate reader (Tecan). Hemolysis rate was then determined according to the following formula:

$${\mathrm{Hemolysis}}\;\left( {\mathrm{\% }} \right) = \left[ {\left( {{\mathrm{OD}}_{{\mathrm{576}}\;{\mathrm{sample}}} - {\mathrm{OD}}_{{\mathrm{576}}\;{\mathrm{blank}}}} \right){\mathrm{/}}\left( {{\mathrm{OD}}_{{\mathrm{576 0}}{\mathrm{.2\% Triton X - 100}}} - {\mathrm{OD}}_{{\mathrm{576 blank}}}} \right)} \right]{\mathrm{ \times 100\% }}.$$

Cytotoxicity on mammalian cells was performed on CHO cells by water-soluble tetrazolium salt-1 (WST-1, Roche) assay via monitoring the absorbance at 450 nm. Tigecycline (16–128 μg mL^−1^) with azidothymidine (0.5 μg mL^−1^) and 1 × 10^4^ cells were simutaneously added in 96-well plates, cultured in DMEM supplemented with 10% heat inactivated FBS at 37 °C in a 5% CO_2_ atmosphere for 24 h, followed by WST-1 tests.

Acute toxicity of the combination of tigecycline with azidothymidine were evaluated in CD-1 mice during 14 days^57^. Mice were randomly divided into two groups (*n* = 10 per group, half male and half female) and intraperitoneally administrated with a single dose of tigecycline (100 mg kg^−1^) in the absence or presence of azidothymidine (10 mg kg^−1^). Survival and body weights of mice were recorded within 14 days after administration.

### Flow cytometry analysis

*E. coli* B3-1 (1 × 10^6^ CFUs) were incubated with PI (10 nM) at room temperature for 15 min in the dark. Then, stained cells were treated with various concentrations of tigecycline, azidothymidine or their combination for 4 h. Subsequently, bacterial cells fluorescence was measured by CytExpert Flow Cytometer (Beckman, USA) and analyzed by CytExpert 2.0 software (Beckman, USA), using excitation wavelength at 535 nm and emission wavelength at 615 nm.

### Resistance study

Sequential culturing of *E. coli* ATCC 25922 in the presence of subinhibitory levels of tigecycline, azidothymidine, or the combination. After 24 h co-incubation, any cultures that grew at higher than the MIC levels were passaged on drug free MHA plates and the MIC was then determined by broth microdilution. This serial passaging was repeated daily for 30 days.

### Conjugation assays

Conjugation determination were estimated using the high-throughput conjugation method previously described^58^. *E. coli* DH5α containing RP4 plasmid and *tet*(X4)-positive *E. coli* S2-1 with high plasmid conjugation frequency were chosen as exconjugant, and *E. coli* EC600 was applied as recipient strains, respectively. Cells were mixed in 1:1 ratio and spotted onto LB-agar plates with varying concentrations of azidothymidine (0–1 μg mL^−1^), meropenem (0.03125 μg mL^−1^), colistin (0.125 μg mL^−1^), or ciprofloxacin (0.125 μg mL^−1^). Mating plates were incubated at 37 °C for 6 h and bacteria were resuspended in M9 broth. The conjugators and conjugation frequencies were measured by bacterial CFU counts. PCR analysis was performed using primers based on forward 5′-TAATTGGTGGTGGACCCGTT-3′ and reverse 5′-CAGCCATTAGCCGGTTTCCA-3′ for partial sequence of *tet*(X4) (product, 588 bp).

### Biochemical factors measurement

Bacteria was preprocessed with same protocols as follows. *E. coli* B3-1 was grown overnight, and the cultures were washed and suspended with 5 mM HEPES. The OD_600_ of bacteria suspension was standardized to 0.5 and different fluorescence dyes were added. After incubated for 30 min at dark, probe-labeled bacterial cells (190 μL) were added to a 96-well plate, and 10 μL of azidothymidine (final concentrations, 0–0.5 μg mL^−1^), tigecycline (final concentrations, 0–64 μg mL^−1^) without or with azidothymidine (final concentrations, 0.25 μg mL^−1^) were added. After incubation for 1 h, fluorescence units were measured on Infinite M200 Microplate reader (Tecan, Mannedorf, Switzerland).

Outer membrane permeability: 1-*N*-phenylnaphthylamine (NPN)^59^ (10 μM) was applied to assess the outer membrane integrity of *E. coli*. Fluorescence units were measured with the excitation wavelength at 350 nm and emission wavelength at 420 nm.

Cell membrane integrity: Fluorescent intensity of 10 nM PI-labeled cells after treatment with drugs was measured with the excitation wavelength at 535 nm and emission wavelength at 615 nm.

Membrane depolarization: 3, 3-dipropylthiadicarbocyanine iodide DiSC_3_(5)^60^ (0.5 μM) was used to monitor bacterial membrane potential. Dissipated membrane potential of *E. coli* in the presence of drugs was determined with excitation wavelength at 622 nm and emission wavelength at 670 nm.

Total ROS: The levels of ROS in *E. coli* were measured with 2′,7′-dichlorodihydrofluorescein diacetate^61^ (DCFH-DA, 10 μM) according to the manufacturer’s instruction (Beyotime). After incubation, the fluorescence intensity was measured with the excitation wavelength at 488 nm and emission wavelength at 525 nm.

### DNA inhibition and RT-PCR analysis

Overnight culture of *E. coli* B3-1 were diluted 1/100 into fresh LB and grown to OD_600_ of 0.1. Then, cells were labeled with 0.25 mCi mL^−1^ of [methyl-^3^H] thymidine to measure the DNA synthesis^62^. After 5 min culture aliquots were treated with varying concentrations of azidothymidine (0–0.5 μg mL^−1^). Ciprofloxacin (0.25 μg mL^−1^, corresponding to 1/2MIC) was used as positive control for inhibition of DNA. At intervals, 100 μL aliquots were taken and precipitated with 6% perchloric acid in a multiscreen filter plate (Millipore). After washing with 0.15 ml of ethanol, the filters were dried and the radioactivity was determined with a liquid scintillation counter (PerkinElmer, WA, USA).

The mRNA levels of *sdhC, mdh, tet*(X4), *recA*, *lexA* and *mazEF* relative to the control genes (16S rRNA) were determined by RT-PCR tests (Supplementary Table 6), which was performed with TB Green *Premix Ex Taq* II (TaKaRa) using 7500 Fast Real-Time PCR System (Applied Biosystem, CA, USA). Thermal cycling was performed by two-step PCR amplification standard procedure with 95 °C for 30 s and 40 cycles of 95 °C for 5 s, 60 °C for 34 s. The fold changes of gene expression were determined using the 2^−ΔΔCt^ method.

### Protein purification and enzyme inhibition assay

Protein expression and enzyme inhibition assays were performed according to previous study^10^. Briefly, the full-length coding sequence of Tet(X3/X4) was cloned into pET28a vector and transformed chemically into *E. coli* BL21 (DE3). The *E. coli* BL21 carrying pET28a was grown to OD_600_ = 0.6 at 37 °C with 200 rpm, followed by the addition of isopropyl-β-d-thiogalactopyrandoside (IPTG, 1 mM). The culture was then incubated overnight at 16 °C with 200 rpm shaking. Subsequently, bacterial cells were collected and lysed by lysis buffer, and protein was purified by His-tag Protein Purification Kit (Beyotime, Shanghai, China). The purity and concentration of the recombinant proteins were measured by sodium dodecyl sulfate polyacrylamide gel electrophoresis and an enhanced BCA protein assay kit (Beyotime, Shanghai, China), respectively.

Ten microgram purified protein were incubated with increasing concentration of azidothymidine for 15 min at 25 °C. Then, the mixture was mixed with equal volume of 3 mM tigecycline. After 1-h incubation, Absorbance at 400 nm^8^ was chosen to monitor the enzyme activity of Tet(X3/X4) proteins in 96-well microtiter plates by Infinite M200 Microplate reader (Tecan).

### In silico docking analysis

Homology modeling of the intact Tet(X3) and Tet(X4) proteins was performed using the SWISS-MODEL (https://swissmodel.expasy.org/)^63^, using the crystal structure of the original Tet(X2)–tigecycline complex (PDB accession number: 4A6N) as template. Molecular docking between Tet(X2), the modeled Tet(X3) and Tet(X4) proteins, and azidothymidine were conducted using the Autodock Vina tool^64^ without the incorporation of water molecules. Interactions between azidothymidine and the residues of the binding sites in Tet(X2/X3/X4) are shown using a two-dimensional diagram by Discovery Studio 4.5.

### Animal study

A 6–8-week-old CD-1 mouse (18–20 g) were obtained from Comparative Medicine Centre of Yangzhou University (Jiangsu, China). Mice were adapted for 1 week prior to bacterial infection. Mice study protocols were performed in accordance with the relevant guidelines and regulations (ID: SCXK-2017–0007). The laboratory animal usage license number is SCXK-2017-0044, certified by Jiangsu Association for Science and Technology.

### Pharmacokinetic analysis

CD-1 female mice were administrated with a single dose of 30 mg kg^−1^ tigecycline and 10 mg kg^−1^ azidothymidine by intraperitoneal injection. No any adverse effects were observed at this dose during 24 h. Plasma samples were taken from 3 mice at each time points (15 min, 30 min, 1 h, 2 h, 4 h, 8 h, 12 h and 24 h). An aliquot of plasma sample (100 μL) was mixed with 3 volumes of acetonitrile (300 μL), vigorously vortexed for 5 min and centrifuged at 12,000 rpm for 10 min. The precipitate is re-extracted with acetonitrile (100 μL) again. Merged supernatants were filtered through a 0.22 μm filter membrane before LC–MS/MS analysis. Then, tigecycline and azidothymidine concentrations in protein-free supernatants were separated on a C18 HPLC column (Agilent) and analyzed by an AB SCIEX 6500 QTRAP^™^ mass spectrometer (Applied Biosystems) in the positive ionization multiple reaction monitoring mode (Supplementary Table 7). The gradient elution system consisting of 0.1% formic acid in water (solvent A) and 0.1% formic acid in acetonitrile (solvent B). Limit of detection, limit of quantitation, recoveries and intra-day relative standard deviation ofthe detection method were displayed in Supplementary Tables 8 and 9. The mean plasma concentrations and the standard deviations at each time point were calculated. Lastly, pharmacokinetic parameters were obtained using a non-compartmental analysis model by WinNonlin 6.4.

### *Galleria mellonella* infection model

*G. mellonella* larvae (Huiyude Biotech Company, Tianjin, China) were randomly divided into four groups (*n* = 10 per group) and injected with 10 µL of *E. coli* B3-1 suspension (1.0 × 10^6^ CFUs) at the right posterior gastropoda. After 2 h post infection, *G. mellonella* larvae were treated with either PBS, tigecycline (32 mg kg^−1^), azidothymidine (1 mg kg^−1^), or the combination of tigecycline plus azidothymidine (32 mg kg^−1^ + 1 mg kg^−1^) at left posterior gastropoda. Survival rates of *G. mellonella* larvae were recorded during 5 days.

### Mouse peritonitis infection model

Female CD-1 mice (*n* = 8 per group) were intraperitoneally infected with *E. coli* B3-1 suspension (3.0 × 10^8^ CFUs per mouse). After 2 h post infection, mice were treated with a single dose of tigecycline (32 mg kg^−1^), azidothymidine (1 mg kg^−1^), or the combination of tigecycline plus azidothymidine (32 mg kg^−1^ + 1 mg kg^−1^) via intraperitoneal injection. Survival rates of treated mice were recorded during 7 days.

### Neutropenic mouse thigh infection model

Female CD-1 mice (*n* = 6 per group) were firstly rendered neutropenic by cyclophosphamide (two doses of 150 and 100 mg kg^−1^ dosed on 4 and 1 days before infection). Then, 100 μL of bacterial suspension (1.5 × 10^5^ CFUs per mouse) were injected into the right thighs of each mouse. After 2 h post infection, mice were treated with either PBS, tigecycline (16 mg kg^−1^), tigecycline (32 mg kg^−1^), azidothymidine (1 mg kg^−1^), azidothymidine (1 mg kg^−1^), or the combination (16 + 1 mg kg^−1^ or 32 + 1 mg kg^−1^) by intraperitoneal injection. At 24 h post infection, mice were euthanized by cervical dislocation. The right thighs were aseptically removed, homogenized, serially diluted, and plated on MHA to count bacterial numbers after incubated at 37 °C for 18 h.

### Statistics and reproducibility

Statistical analysis was performed using GraphPad Prism 7 software. All data were obtained from at least three independent experiments and presented as means ± SD. In the in vitro studies, unpaired *t* test between two groups or one-way ANOVA among multiple groups were used to calculate *P* values. In animal studies, significance of bacterial load in neutropenic mouse thigh infection experiment and survival rates in mouse peritonitis-sepsis were analyzed by Mann–Whitney *U* test or log-rank (Mantel–Cox) test, respectively.

### Reporting summary

Further information on research design is available in the Nature Research Reporting Summary linked to this article.

## Supplementary information

Supplementary Information

Description of Additional Supplementary Files

Supplementary Data 1

Reporting Summary

## Data Availability

Source data underlying Figs. 2–5 and Supplementary Fig. 2, Supplementary Fig. 3, Supplementary Fig. 5–7 are in the Supplementary Data 1 file. All other data are available upon request from the corresponding author.

## References

[1] Walsh TR, Weeks J, Livermore DM, Toleman MA (2011). Dissemination of NDM-1 positive bacteria in the New Delhi environment and its implications for human health: an environmental point prevalence study. Lancet Infect. Dis..

[2] Liu Y-Y (2016). Emergence of plasmid-mediated colistin resistance mechanism MCR-1 in animals and human beings in China: a microbiological and molecular biological study. Lancet Infect. Dis..

[3] Pankey GA (2005). Tigecycline. J. Antimicrob. Chemother..

[4] Stein GE, Babinchak T (2013). Tigecycline: an update. Diagn. Microbiol. Infect. Dis..

[5] Whittle G, Hund BD, Shoemaker NB, Salyers AA (2001). Characterization of the 13-kilobaseermF region of the Bacteroides conjugative transposon CTnDOT. Appl. Environ. Microbiol..

[6] Sun J (2019). Plasmid-encoded *tet*(X) genes that confer high-level tigecycline resistance in *Escherichia coli*. Nat. Microbiol..

[7] He T (2019). Emergence of plasmid-mediated high-level tigecycline resistance genes in animals and humans. Nat. Microbiol..

[8] Moore IF, Hughes DW, Wright GD (2005). Tigecycline is modified by the flavin-dependent monooxygenase TetX. Biochemistry.

[9] Volkers G, Palm GJ, Weiss MS, Wright GD, Hinrichs W (2011). Structural basis for a new tetracycline resistance mechanism relying on the TetX monooxygenase. FEBS Lett..

[10] Wangrong Y (2004). TetX is a flavin-dependent monooxygenase conferring resistance to tetracycline antibiotics. J. Biol. Chem..

[11] Liu Y, Ding S, Shen J, Zhu K (2019). Nonribosomal antibacterial peptides that target multidrug-resistant bacteria. Nat. Prod. Rep..

[12] Payne DJ, Gwynn MN, Holmes DJ, Pompliano DL (2007). Drugs for bad bugs: confronting the challenges of antibacterial discovery. Nat. Rev. Drug. Discov..

[13] Liu Y, Li R, Xiao X, Wang Z (2019). Antibiotic adjuvants: an alternative approach to overcome multi-drug resistant Gram-negative bacteria. Crit. Rev. Microbiol..

[14] Mullard A (2014). New drugs cost US $2.6 billion to develop. Nat. Rev. Drug. Discov..

[15] Robbel L, Marahiel MA (2010). Daptomycin, a bacterial lipopeptide synthesized by a nonribosomal machinery. J. Biol. Chem..

[16] Wright GD (2016). Antibiotic adjuvants: rescuing antibiotics from resistance. Trends Microbiol..

[17] King AM (2014). Aspergillomarasmine A overcomes metallo-β-lactamase antibiotic resistance. Nature.

[18] Garcia-Fernandez E (2017). Membrane microdomain disassembly inhibits MRSA antibiotic resistance. Cell.

[19] Stokes JM (2017). Pentamidine sensitizes Gram-negative pathogens to antibiotics and overcomes acquired colistin resistance. Nat. Microbiol.

[20] Lyu Y (2019). Design of Trp-rich dodecapeptides with broad-spectrum antimicrobial potency and membrane-disruptive mechanism. J. Med. Chem..

[21] Zipperer A (2016). Human commensals producing a novel antibiotic impair pathogen colonization. Nature.

[22] Hancock R, Wong P (1984). Compounds which increase the permeability of the *Pseudomonas aeruginosa* outer membrane. Antimicrob. Agents Chemother..

[23] McGrath DM (2013). Mechanism of action and initial evaluation of a membrane active all-*D*-enantiomer antimicrobial peptidomimetic. Proc. Natl Acad. Sci. USA.

[24] Mc Dermott PF, Walker RD, White DG (2003). Antimicrobials: modes of action and mechanisms of resistance. Int. J. Toxicol..

[25] Harrison E, Brockhurst MA (2012). Plasmid-mediated horizontal gene transfer is a coevolutionary process. Trends Microbiol..

[26] Tyers M, Wright GD (2019). Drug combinations: a strategy to extend the life of antibiotics in the 21st century. Nat. Rev. Microbiol..

[27] Tomas C, Ray AS (2010). Nucleoside and nucleotide HIV reverse transcriptase inhibitors: 25 years after zidovudine. Antivir. Res..

[28] Giles C (1991). Clinical pharmacokinetics of parenterally administered danofloxacin in cattle. J. Vet. Pharmacol. Ther..

[29] Yelin I, Kishony R (2018). Antibiotic resistance. Cell.

[30] Kupferschmidt K (2016). Resistance fighter. Science.

[31] O’Neill J. *Tackling Drug-Resistant Infections Globally: Final Report and Recommendations*. (The Review on Microbial Resistance, 2016).

[32] Woodford N, Turton JF, Livermore DM (2011). Multiresistant Gram-negative bacteria: the role of high-risk clones in the dissemination of antibiotic resistance. FEMS Microbiol. Rev..

[33] Karageorgopoulos DE, Falagas ME (2008). Current control and treatment of multidrug-resistant *Acinetobacter baumannii* infections. Lancet Infect. Dis..

[34] Rodríguez-Baño J, Gutiérrez-Gutiérrez B, Machuca I, Pascual A (2018). Treatment of infections caused by extended-spectrum-beta-lactamase-, AmpC-, and carbapenemase-producing Enterobacteriaceae. Clin. Microbiol. Rev..

[35] Brochado AR (2018). Species-specific activity of antibacterial drug combinations. Nature.

[36] Ng SMS (2018). Repurposing Zidovudine in combination with tigecycline for treating carbapenem-resistant Enterobacteriaceae infections. Eur. J. Clin. Microbiol. Infect. Dis..

[37] Vogt MW (1987). Ribavirin antagonizes the effect of azidothymidine on HIV replication. Science.

[38] Fischl MA (1987). The efficacy of azidothymidine (AZT) in the treatment of patients with AIDS and AIDS-related complex. N. Engl. J. Med..

[39] Elwell LP (1987). Antibacterial activity and mechanism of action of 3’-azido-3’-deoxythymidine (BW A509U). Antimicrob. Agents Chemother..

[40] Monno R (1997). In vitro antimicrobial properties of azidothymidine (AZT). Acta Microbiol. Immunol. Hung..

[41] Falagas ME (2019). Synergistic activity of colistin with azidothymidine against colistin-resistant *Klebsiella pneumoniae* clinical isolates collected from inpatients in Greek hospitals. Int. J. Antimicrob. Agents.

[42] Wambaugh MA, Shakya VPS, Lewis AJ (2017). High-throughput identification and rational design of synergistic small-molecule pairs for combating and bypassing antibiotic resistance. PLoS Biol..

[43] Cooper DL, Lovett ST (2011). Toxicity and tolerance mechanisms for azidothymidine, a replication gap-promoting agent, in *Escherichia coli*. DNA repair.

[44] Lam SJ (2016). Combating multidrug-resistant Gram-negative bacteria with structurally nanoengineered antimicrobial peptide polymers. Nat. Microbiol..

[45] Erental A, Sharon I, Engelberg-Kulka H (2012). Two programmed cell death systems in *Escherichia coli*: an apoptotic-like death is inhibited by the *mazEF*-mediated death pathway. PLoS Biol..

[46] Bayles KW (2014). Bacterial programmed cell death: making sense of a paradox. Nat. Rev. Microbiol..

[47] Erental A, Kalderon Z, Saada A, Smith Y, Engelberg-Kulka H (2014). Apoptosis-like death, an extreme SOS response in *Escherichia coli*. mBio.

[48] McGuirk PR (1992). Synthesis and structure-activity relationships of 7-diazabicycloalkylquinolones, including danofloxacin, a new quinolone antibacterial agent for veterinary medicine. J. Med. Chem..

[49] Singh R, Lillardab JW (2009). Nanoparticle-based targeted drug delivery. Exp. Mol. Pathol..

[50] Allen TM, Cullis PR (2013). Liposomal drug delivery systems: from concept to clinical applications. Adv. Drug Deliv. Rev..

[51] Clinical Lab Standards Institute. Performance standards for antimicrobial susceptibility testing. *CLSI* (2016).

[52] Wang R (2018). Bismuth antimicrobial drugs serve as broad-spectrum metallo-β-lactamase inhibitors. Nat. Commun..

[53] Liu Y, Yang K, Jia Y, Wang Z (2019). Repurposing peptidomimetic as potential inhibitor of New Delhi Metallo-β-lactamases in Gram-negative bacteria. ACS Infect. Dis..

[54] Macnair CR (2018). Overcoming *mcr−1* mediated colistin resistance with colistin in combination with other antibiotics. Nat. Commun..

[55] Liu Y, Song M, Ding S, Zhu K (2019). Discovery of linear low-cationic peptides to target methicillin-resistant *Staphylococcus aureus in vivo*. ACS Infect. Dis..

[56] Liu Y, Ding S, Dietrich R, Märtlbauer E, Zhu K (2017). A biosurfactant-inspired heptapeptide with improved specificity to kill MRSA. Angew. Chem. Int. Ed..

[57] Lorke D (1983). A new approach to practical acute toxicity testing. Arch. Toxicol..

[58] Xie M, Li R, Liu Z, Chan EWC, Chen S (2018). Recombination of plasmids in a carbapenem-resistant NDM-5-producing clinical *Escherichia coli* isolate. J. Antimicrob. Chemother..

[59] Ejim L (2011). Combinations of antibiotics and nonantibiotic drugs enhance antimicrobial efficacy. Nat. Chem. Biol..

[60] Hamamoto H (2015). Lysocin E is a new antibiotic that targets menaquinone in the bacterial membrane. Nat. Chem. Biol..

[61] Chen X, Zhong Z, Xu Z, Chen L, Wang Y (2010). 2′,7′-Dichlorodihydrofluorescein as a fluorescent probe for reactive oxygen species measurement: forty years of application and controversy. Free Radic. Res..

[62] Ling LL (2015). A new antibiotic kills pathogens without detectable resistance. Nature.

[63] Waterhouse A (2018). SWISS-MODEL: homology modelling of protein structures and complexes. Nucleic Acids Res..

[64] Trott O, Olson AJ (2010). AutoDock Vina: improving the speed and accuracy of docking with a new scoring function, efficient optimization, and multithreading. J. Comput. Chem..

