# Supplementary material for: Anti-HIV agent azidothymidine decreases Tet(X)-mediated bacterial resistance to tigecycline in Escherichia coli
Source: Commun Biol. 2020 Apr 3;3:162. doi: 10.1038/s42003-020-0877-5 (PMC7125129; doi:10.1038/s42003-020-0877-5)
Supplement: Supplementary file 1 — Supplementary Information [file 42003_2020_877_MOESM1_ESM.pdf]

Supplementary Figures

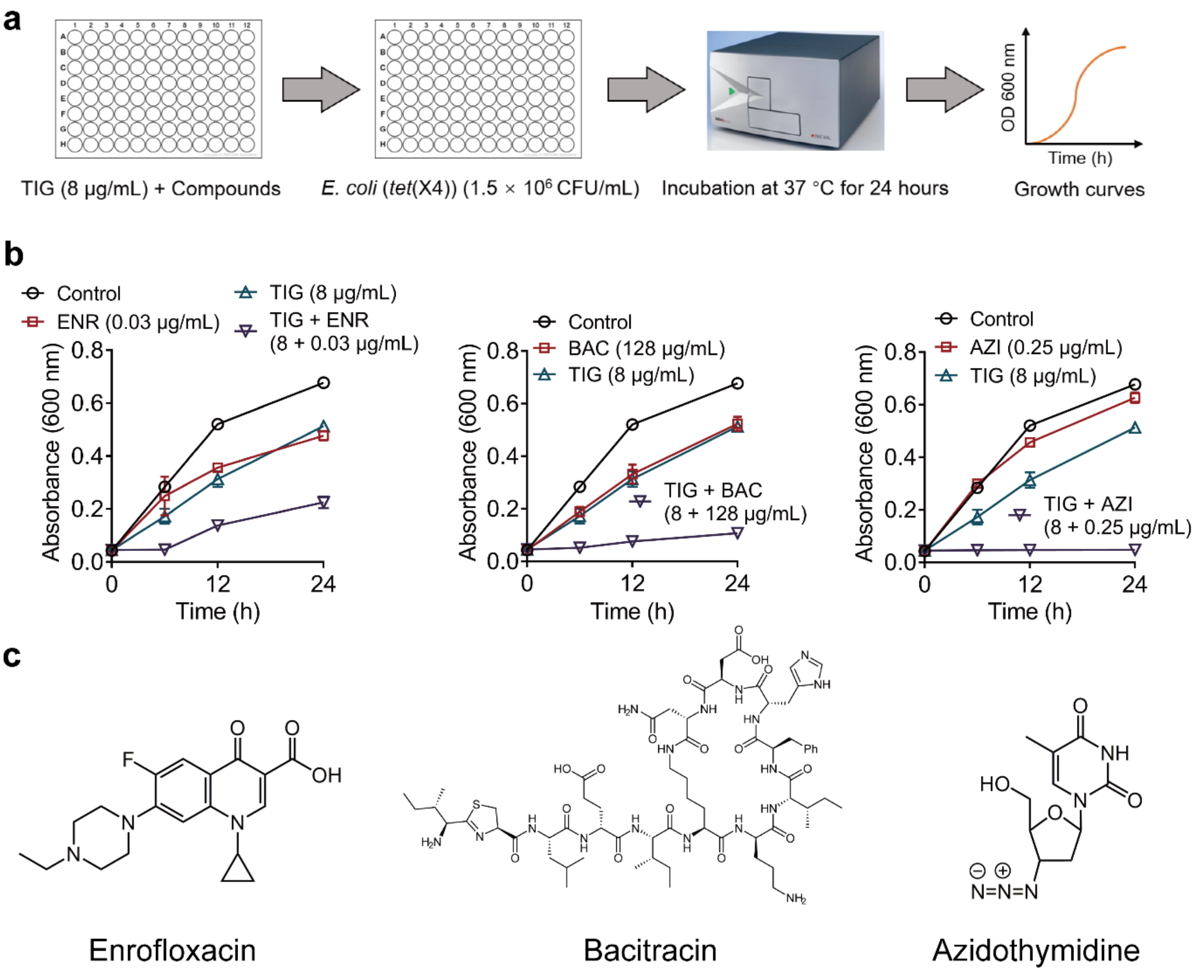

Supplementary Fig. 1 Primary screening of tigecycline adjuvants against Tet(X4)-expression *E. coli* B3-1.

(a) Scheme of identification of tigecycline adjuvants. **(b)** Growth curves of *E. coli* B2 in the presence of one quarter of MIC of tigecycline, compounds alone or combination. TIG, tigecycline. ENR, enrofloxacin. BAC, bacitracin. AZI, azidothymidine. **(c)** Chemical structures of three adjuvant candidates.

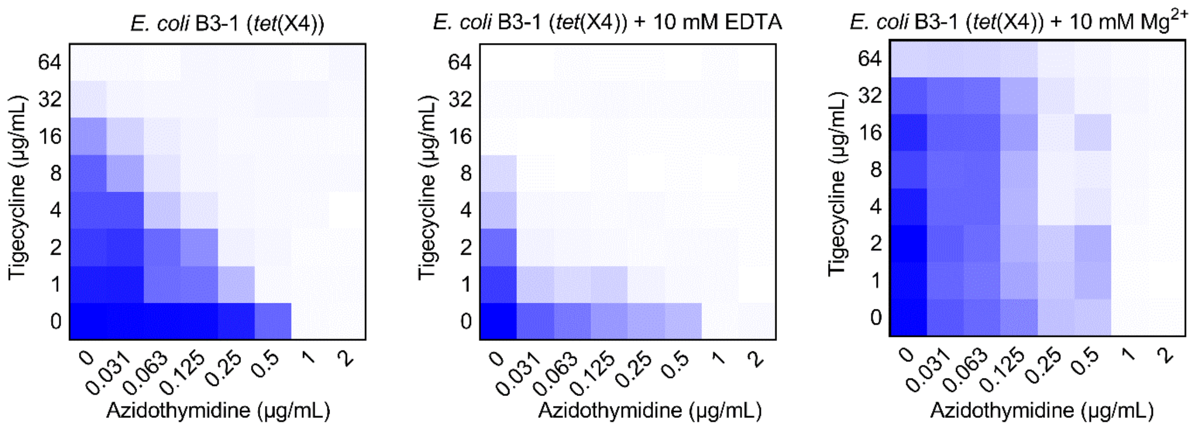

Supplementary Fig. 2 Effect of 10 mM Mg^{2+} or EDTA on the potentiation activity of azidothymidine with tigecycline.

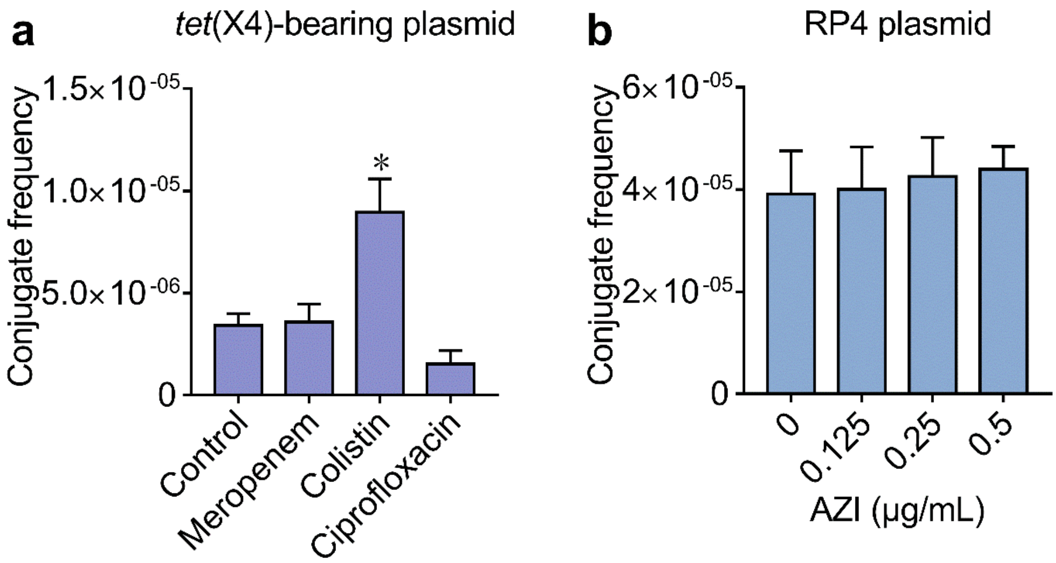

Supplementary Fig. 3 Effect of drugs on the conjugation of *tet*(X4)-bearing plasmid and RP4 plasmid.

(a) Meropenem and ciprofloxacin have no effect on horizontal transfer of *tet*(X4), while colistin mildly promotes the conjugation. One quarter of MIC concentrations of meropenem (0.03125 μg/mL), colistin (0.125 μg/mL) and ciprofloxacin (0.125 μg/mL) were used.

(b) Azidothymidine has no effect on conjugation frequency of conjugative plasmid RP4.

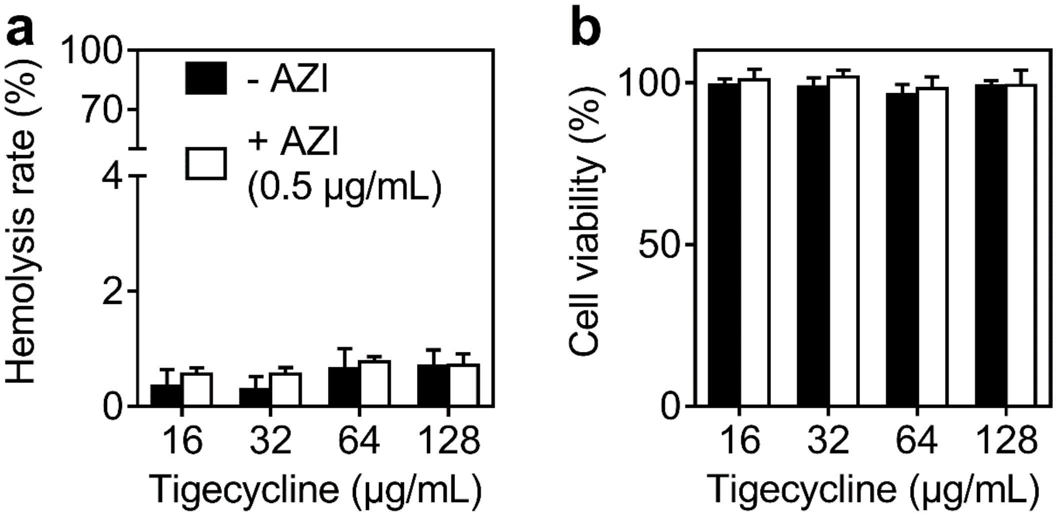

Supplementary Fig. 4 Safety assessment of the combination of tigecycline and azidothymidine.

(a) Hemolytic activity of tigecycline in the absence or presence of sub-MIC of azidothymidine (0.5 µg/mL) to the RBCs.

(b) Cytotoxicity of CHO cells treated with tigecycline with or without azidothymidine (0.5 µg/mL) was evaluated by WST-1 assay.

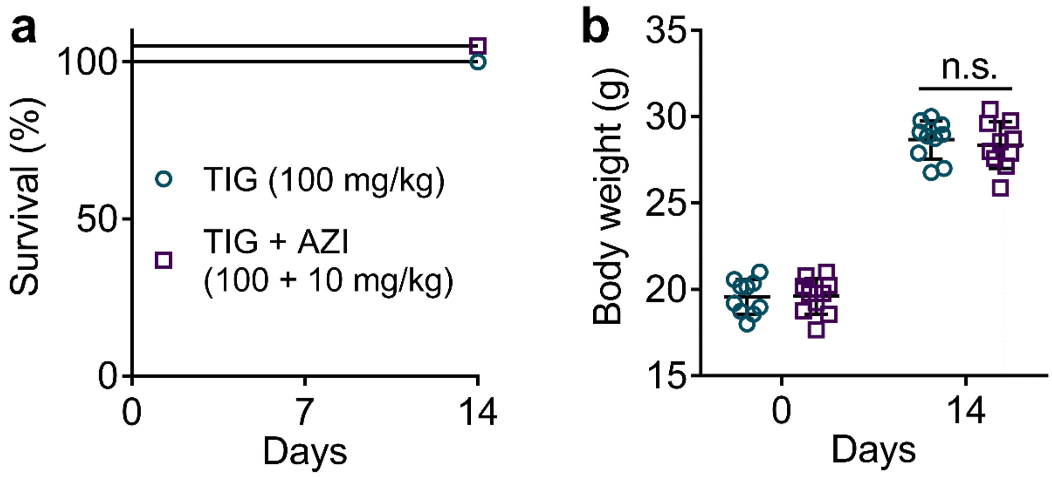

Supplementary Fig. 5 Acute toxicity of tigecycline and the combination of tigecycline plus azidothymidine in mice.

Survival rates **(a)** and body weights **(b)** of mice (n = 10 per group) after a single high dose of tigecycline (100 mg/kg) without or with azidothymidine (10 mg/kg) via intraperitoneal injection. n.s., not significant, determined by Mann-Whitney U test.

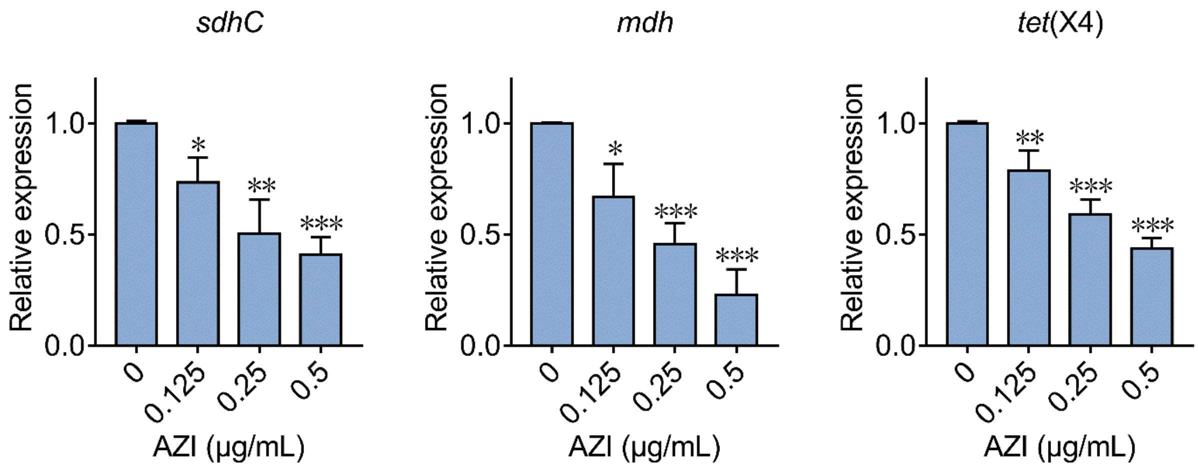

Supplementary Fig. 6 Azidothymidine inhibits expression of *sdhC*, *mdh* and *tet*(X4) by RT-PCR.

All data were expressed as mean ± SD. * $P < 0.05$, **$P < 0.01$, ***$P < 0.001$, determined by non-parametric one-way ANOVA.

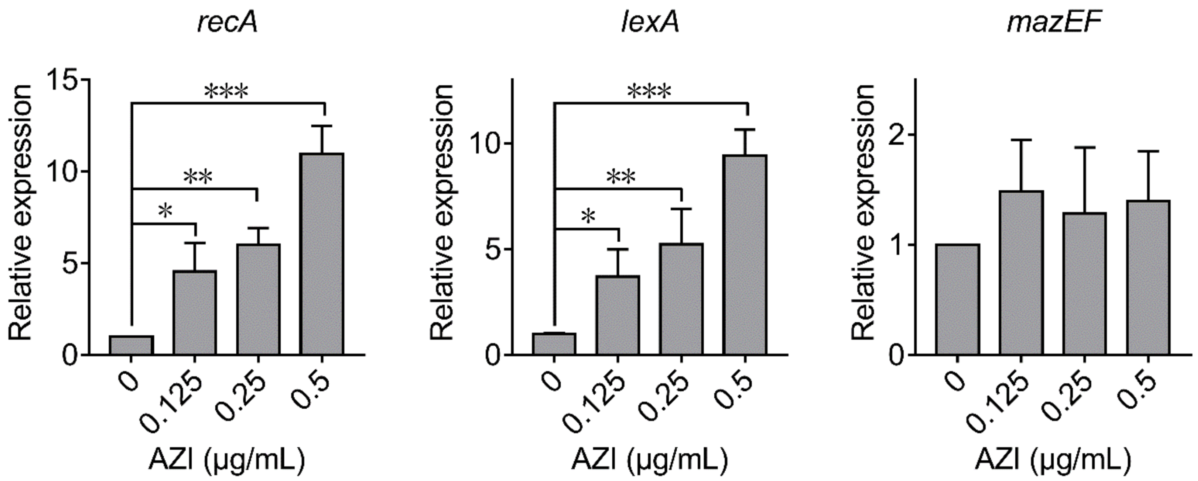

Supplementary Fig. 7 Azidothymidine induces DNA repair in *E. coli* via upregulating the expression of *rec*A and *lex*A. All data were expressed as mean ± SD as indicated by the error bars (n = 3) of the fold increase in RNA levels as determined by real-time PCR from untreated cells.

*$P < 0.05$, **$P < 0.01$, ***$P < 0.001$, determined by non-parametric one-way ANOVA.

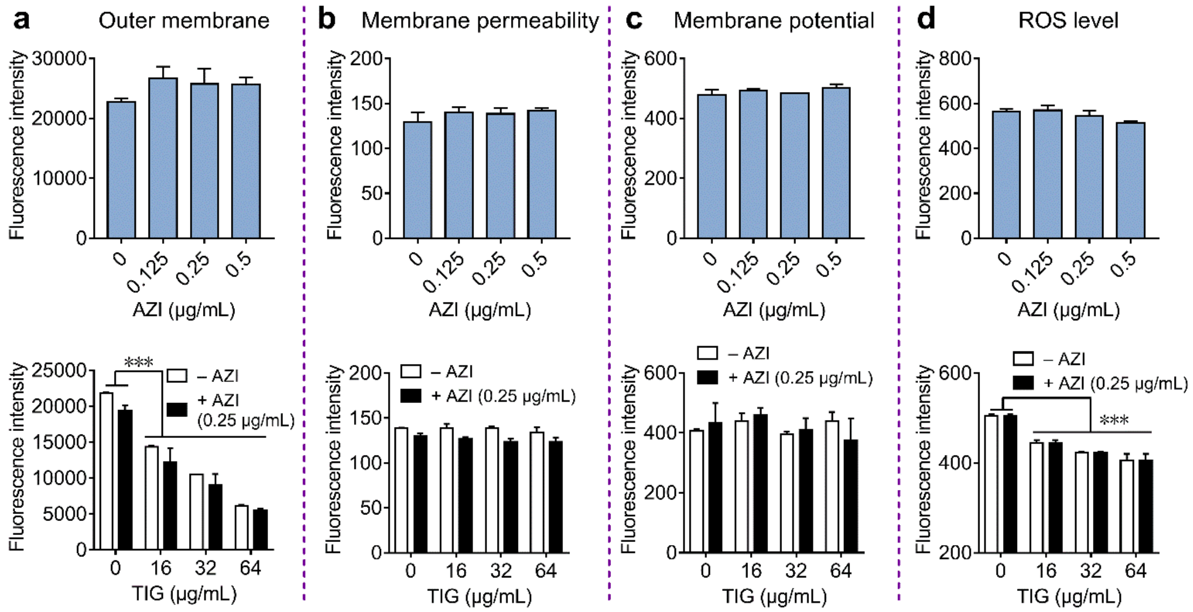

Supplementary Fig. 8 Potentiation activity of azidothymidine with tigecycline is independent of membrane disruption.

Outer membrane integrity **(a)**, membrane permeability **(b)**, membrane potential **(c)** and ROS level **(d)** of *E. coli* B3-1 with treatment either azidothymidine or tigecycline, or a combination of tigecycline plus azidothymidine for 1 h, probed by 1-*N*-phenylnaphthylamine (NPN), propidium iodide (PI), $DiSC_3(5)$ and 2′,7′-dichlorofluorescein diacetate (DCFH-DA), respectively.

a

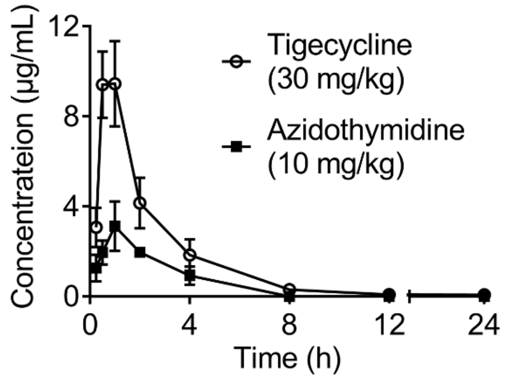

b

Parameters	Tigecycline	Azidothymidine
AUC_{last} (h×μg/mL)	25.43±0.71	9.15±3.16
Cmax (μg/mL)	10.97±2.12	3.45±1.37
Tmax (h)	0.83±0.29	1.33±0.58
Vz_F	6.46±0.43	7.04±9.04
Cl_F	1.18±0.03	1.21±0.52
HL_Lambda_z (h)	3.80±0.26	3.15±3.08
Lambda_z (h)	0.18±0.01	0.43±0.37
MRTINF_obs (h)	3.18±0.24	2.20±0.36

Supplementary Fig. 9 Pharmacokinetic analysis of tigecycline and azidothymidine in mice.

(**a**) The mean plasma concentrations of tigecycline and azidothymidine after a single i.p. injection of 30 mg/kg tigecycline and 10 mg/kg azidothymidine (3 mice per time point). All data were presented as means ± SD.

(b) Pharmacokinetic parameters of tigecycline and azidothymidine calculated with a non-compartmental analysis model based on WinNonlin. AUC_{last}: area under the plasma concentration-time curve from time 0 to 24 h; Cmax: maximal plasma concentration; Tmax: time to maximal plasma concentration; Vz_F: the volume of distribution estimated based on total AUC; Cl_F: total body clearance; HL_Lambda_z: terminal half-life of the drug; Lambda_z: the elimination rate constant estimated from the regression line representing the terminal phase of the concentration-time profile; MRTINF_obs: the mean residence time from the first sampling time extrapolated to infinity based on the last observed concentration.

Supplementary Tables

Supplementary Table 1 Interaction between compounds with tigecycline against *E. coli* B3-1, related to Figure 1.

Compounds	Inhibition rate (%)[#]	Interaction
Cefquinome	4.35 ± 1.58	Indifference
Ceftiofur	2.35 ± 2.50	Indifference
Meropenem	3.58 ± 3.04	Indifference
Aztreonam	2.68 ± 1.75	Indifference
Streptomycin	9.34 ± 0.54	Indifference
Kanamycin	5.68 ± 3.01	Indifference
Spectinomycin	34.87 ± 6.24	Indifference
Tetracycline	-5.35 ± 1.25	Antagonism
Doxycycline	7.24 ± 1.35	Indifference
Minocycline	-10.21 ± 3.01	Antagonism
Florfenicol	4.68 ± 2.87	Indifference
Enrofloxacin	70.16 ± 5.14	Synergy
Sulfamethoxazole	-10.24 ± 3.01	Antagonism
Colistin	9.14 ± 1.24	Indifference
Rifampicin	34.21 ± 5.68	Indifference
Bacitracin	82.8 ± 1.89	Synergy
Metformin	7.25 ± 2.08	Indifference
Melatonin	3.54 ± 1.71	Indifference
Tryptophan	4.45 ± 0.89	Indifference
Stigmasterol	29.74 ± 4.14	Indifference
Lovastatin	-2.56 ± 1.34	Antagonism
Azidothymidine	95.24 ± 1.36	Synergy
Ascorbic acid	2.65 ± 1.87	Indifference
Indole	30.68 ± 3.68	Indifference
Aspirin	-5.68 ± 2.71	Antagonism
Vanillin	-10.56 ± 5.38	Antagonism

[#]Data are representative of three independent experiments ± SD.

Supplementary Table 2 Bacterial strains used in this study.

Organism and genotypes	Source
**Gram-positive pathogens**	In this study
Staphylococcus aureus 29213	ATCC
S. aureus 215 (LZD^R + *cfr*)	In this study
MRSA T144	In this study
MRSA 1518	In this study
Enterococcus faecalis VRE A4	In this study
**Gram-negative pathogens**	In this study
Escherichia coli ATCC 25922	ATCC
E. coli C3 (NDM-1)	In this study
E. coli G6 (NDM-5)	In this study
E. coli B2 (NDM-5 + MCR-1)	In this study
E. coli B3-1 (*tet*(X4))	In this study
E. coli B9-1 (*tet*(X4))	In this study
E. coli B8-1 (*tet*(X4))	In this study
E. coli W7-1 (*tet*(X4))	In this study
E. coli P3-1 (*tet*(X4))	In this study
E. coli P10-1 (*tet*(X4))	In this study
E. coli F168-1 (*tet*(X4))	In this study
E. coli F14 (*tet*(X4))	In this study
E. coli F12-1 (*tet*(X4))	In this study
E. coli P12-1 (*tet*(X4))	In this study
E. coli T29 (*tet*(X4))	In this study
E. coli T18-1 (*tet*(X4))	In this study
E. coli W4-1 (*tet*(X4))	In this study
E. coli W6-1 (*tet*(X4))	In this study
E. coli W8-1 (*tet*(X4))	In this study
E. coli D5-1 (*tet*(X4))	In this study
E. coli S2-1 (*tet*(X4))	In this study
E. coli S3-1 (*tet*(X4))	In this study
E. coli S5-1 (*tet*(X4))	In this study
E. coli S6-2 (*tet*(X4))	In this study
E. brevis S1-3 (*tet*(X3))	In this study
E. fergusonnii 1C4-6 (*tet*(X4))	In this study
Proteus penneri 2F1-3 (*tet*(X4))	In this study
Shigella 1F25-27 (*tet*(X4))	In this study
Salmonella enterica 13076	ATCC

LZD^R, linezolid resistant; MRSA, methicillin-resistant *Staphylococcus aureus*; VRE, vancomycin resistant Enterococcus.

Supplementary Table 3 Antibacterial spectrum of azidothymidine.

Strains	MIC (µg/mL)
**Gram-positive pathogens**	
Staphylococcus aureus ATCC 29213	>16
S. aureus 215 (LZD^{R} + *cfr*)	>16
MRSA T144	>16
MRSA 1518	>16
Enterococcus faecalis VRE A4	>16
**Gram-negative pathogens**	
Escherichia coli ATCC 25922	2
E. coli C3 (NDM-1)	>16
E. coli G6 (NDM-5)	4
E. coli B2 (NDM-5 + MCR-1)	>16
E. coli B3-1 (*tet*(X4))	1
E. coli B9-1 (*tet*(X4))	1
E. coli B8-1 (*tet*(X4))	0.5
E. coli W7-1 (*tet*(X4))	2
E. coli P3-1 (*tet*(X4))	0.5
E. coli P10-1 (*tet*(X4))	0.25
E. coli F168-1 (*tet*(X4))	0.5
E. coli F14 (*tet*(X4))	8
E. coli F12-1 (*tet*(X4))	1
E. coli P12-1 (*tet*(X4))	1
E. coli T29 (*tet*(X4))	2
E. coli T18-1 (*tet*(X4))	0.125
E. coli W4-1 (*tet*(X4))	0.5
E. coli W6-1 (*tet*(X4))	1
E. coli W8-1 (*tet*(X4))	2
E. coli D5-1 (*tet*(X4))	1
E. coli S2-1 (*tet*(X4))	0.25
E. coli S3-1 (*tet*(X4))	0.25
E. coli S5-1 (*tet*(X4))	0.5
E. coli S6-2 (*tet*(X4))	0.5
E. brevis S1-3 (*tet*(X3))	>32
E. fergusonnii 1C4-6 (*tet*(X4))	8
Proteus penneri 2F1-3 (*tet*(X4))	8
Shigella 1F25-27 (*tet*(X4))	2
Salmonella enterica ATCC 13076	0.25

Supplementary Table 4 Synergistic activity of azidothymidine and tigecycline against conjugator.

Pathogens	MIC[a] (μg/mL)	MIC[b] (μg/mL)	FIC index	Potentiation (fold)[c]
E. coli EC600 (Recipients)	1	0.25	0.375	4
E. coli EC600-*tet*(X4) (Transconjugants)	32	1	0.094	32

[a/b] MICs of tigecycline in the absence or presence of non-lethal concentration of azidothymidine.

[c] Degree of tigecycline potentiation in the presence of non-lethal concentration of azidothymidine.

Supplementary Table 5 Antibacterial activity of drugs against *E. coli* B3-1 in the presence of EDTA or Mg^{2+} (MIC, µg/mL).

Drugs	MHB	MHB + EDTA (10 mM)	MHB + Mg^{2+} (10 mM)
Tigecycline	32	8	128
Azidothymidine	1	1	1

Supplementary Table 6 Primers for RT-PCR analysis in this study.

Genes	Primers (5'-3')
sdhC	CCTTACCGCTCTGGCGTATC
	TGCGAGAAGTGAAAGCACGA
mdh	CGGTTATTGGCGGTCACTCT
	CGTTCTGGATGCGTTTGGTC
tet(X4)	TGGGACGAACGCTACAAAGA
	GAGGCATCAAATGAGCAGCA
recA	AGATCCTCTACGGCGAAGGT
	CCTGCTTTCTCGATCAGCTT
lexA	GACTTGCTGGCAGTGCATAA
	TCAGGCGCTTAACGGTAACT
16SrRNA	TGTAGCGGTGAAATGCGTAGA
	CACCTGAGCGTCAGTCTTCGT

Supplementary Table 7 MRM parameters for the determination of tigecycline and azidothymidine by LC-MS/MS.

Compounds	Precursor ions (*m/z*)	Daughter ions (*m/z*)	DP (V)	CE (eV)
Tigecycline	586.4	569.8*	116	29.52
		513.6	116	34.39
Azidothymidine	268.0	127.0*	32	14.64
		202.9	66	20.75

*, quantitative ion; DP, declustering potential; CE, collision energy.

Supplementary Table 8 Regression equation, LOD and LOQ of tigecycline and azidothymidine.

Compounds	Regression equation	R^2	LOD (ng/mL)	LOQ (ng/mL)
Tigecycline	Y = 377392X + 114463	0.9954	3.0	10.0
Azidothymidine	Y = 3E+06X – 5192.7	0.9974	2.5	8.0

LOD, limit of detection; LOQ, limit of quantity.

Supplementary Table 9 Recovery, accuracy and precision of tigecycline and Azidothymidine in plasma samples by LC-MS/MS.

Compounds	Spiked level (ng/mL)	Recovery (%)	Intra-RSD (%) (n = 6)
Tigecycline	10	87.87	3.4
	100	88.13	6.8
	1000	83.87	7.2
Azidothymidine	8	82.26	1.2
	100	89.12	6.7
	1000	82.43	7.3
