# Supplementary material for: Anti-HIV agent azidothymidine decreases Tet(X)-mediated bacterial resistance to tigecycline in Escherichia coli
Source: Commun Biol. 2020 Apr 3;3:162. doi: 10.1038/s42003-020-0877-5 (PMC7125129; doi:10.1038/s42003-020-0877-5)
Supplement: Supplementary file 2 — Description of Additional Supplementary Files [file 42003_2020_877_MOESM2_ESM.pdf]

Description of additional supplementary files

Supplementary Data 1 contains the data underlying Figure 2, Figure 3, Figure 4, Figure 5, Supplementary Figure 2, Supplementary Figure 3, Supplementary Figure 5, Supplementary Figure 6 and Supplementary Figure 7.
