# Supplementary material for: Anti-HIV agent azidothymidine decreases Tet(X)-mediated bacterial resistance to tigecycline in Escherichia coli
Source: Commun Biol. 2020 Apr 3;3:162. doi: 10.1038/s42003-020-0877-5 (PMC7125129; doi:10.1038/s42003-020-0877-5)
Supplement: Supplementary file 4 — Reporting Summary [file 42003_2020_877_MOESM4_ESM.pdf]

nature research

Corresponding author(s): Yuan Liu and Zhiqiang Wang

Last updated by author(s): Feb 19, 2020

Reporting Summary

Nature Research wishes to improve the reproducibility of the work that we publish. This form provides structure for consistency and transparency in reporting. For further information on Nature Research policies, see Authors & Referees and the Editorial Policy Checklist.

Statistics

For all statistical analyses, confirm that the following items are present in the figure legend, table legend, main text, or Methods section.

n/a	Confirmed	
☐	☒	The exact sample size (*n*) for each experimental group/condition, given as a discrete number and unit of measurement
☐	☒	A statement on whether measurements were taken from distinct samples or whether the same sample was measured repeatedly
☐	☒	The statistical test(s) used AND whether they are one- or two-sided<br>*Only common tests should be described solely by name; describe more complex techniques in the Methods section.*
☐	☒	A description of all covariates tested
☐	☒	A description of any assumptions or corrections, such as tests of normality and adjustment for multiple comparisons
☐	☒	A full description of the statistical parameters including central tendency (e.g. means) or other basic estimates (e.g. regression coefficient) AND variation (e.g. standard deviation) or associated estimates of uncertainty (e.g. confidence intervals)
☐	☒	For null hypothesis testing, the test statistic (e.g. *F*, *t*, *r*) with confidence intervals, effect sizes, degrees of freedom and *P* value noted<br>*Give P values as exact values whenever suitable.*
☒	☐	For Bayesian analysis, information on the choice of priors and Markov chain Monte Carlo settings
☒	☐	For hierarchical and complex designs, identification of the appropriate level for tests and full reporting of outcomes
☒	☐	Estimates of effect sizes (e.g. Cohen's *d*, Pearson's *r*), indicating how they were calculated

Our web collection on statistics for biologists contains articles on many of the points above.

Software and code

Policy information about availability of computer code

Data collection	Fluorescence and absorbance plate reader data were collected using Infinite M200 Microplate reader (Tecan).
Data analysis	GraphPad Prism versions 7.00.159<br>Microsoft Excel 2019

For manuscripts utilizing custom algorithms or software that are central to the research but not yet described in published literature, software must be made available to editors/reviewers. We strongly encourage code deposition in a community repository (e.g. GitHub). See the Nature Research guidelines for submitting code & software for further information.

Data

Policy information about availability of data

All manuscripts must include a data availability statement. This statement should provide the following information, where applicable:

- Accession codes, unique identifiers, or web links for publicly available datasets
- A list of figures that have associated raw data
- A description of any restrictions on data availability

All data are available in the supplemental information and/or from the corresponding author upon request.

Field-specific reporting

Please select the one below that is the best fit for your research. If you are not sure, read the appropriate sections before making your selection.

☒ Life sciences ☐ Behavioural & social sciences ☐ Ecological, evolutionary & environmental sciences

Life sciences study design

All studies must disclose on these points even when the disclosure is negative.

Sample size	In vivo animal model: No specific statistical methods were used to predetermine the sample size; however, we use the animal number (n≥6) of statistical significance for the testing, and all data were presented as means ± SD.
Data exclusions	No exclusions.
Replication	Primary data were generated from at least 3 independent replicates.
Randomization	In vivo efficacy: CD-1 female mice were adapted for one week prior to infection. All animals were grouped randomly.
Blinding	Investigators were blinded as for group allocation during data collection and analysis.

Reporting for specific materials, systems and methods

We require information from authors about some types of materials, experimental systems and methods used in many studies. Here, indicate whether each material, system or method listed is relevant to your study. If you are not sure if a list item applies to your research, read the appropriate section before selecting a response.

Materials & experimental systems

n/a	Involved in the study	
☒	☐	Antibodies
☐	☒	Eukaryotic cell lines
☒	☐	Palaeontology
☐	☒	Animals and other organisms
☒	☐	Human research participants
☒	☐	Clinical data

Methods

n/a	Involved in the study	
☒	☐	ChIP-seq
☐	☒	Flow cytometry
☒	☐	MRI-based neuroimaging

Eukaryotic cell lines

Policy information about cell lines

Cell line source(s)	Chinese Hamster Ovary (CHO) cells, American Type Culture Collection (Manassas, VA, USA).
Authentication	None of the cell lines were authenticated in our lab.
Mycoplasma contamination	The cell lines tested negative for mycoplasma contamination.
Commonly misidentified lines (See ICLAC register)	None.

Animals and other organisms

Policy information about studies involving animals; ARRIVE guidelines recommended for reporting animal research

Laboratory animals	Female CD-1 mice, 6-8 weeks old.
Wild animals	The study did not involve wild animals.
Field-collected samples	The study did not involve samples collected from the field.
Ethics oversight	The animal use procedures were approved by the Animal Research Committees of Yangzhou University (YZU). All animal studies were conducted in accordance with YZU Institutional Animal Welfare and Ethics guidelines.

Note that full information on the approval of the study protocol must also be provided in the manuscript.

Flow Cytometry

Plots

Confirm that:

☒ The axis labels state the marker and fluorochrome used (e.g. CD4-FITC).

☒ The axis scales are clearly visible. Include numbers along axes only for bottom left plot of group (a 'group' is an analysis of identical markers).

☒ All plots are contour plots with outliers or pseudocolor plots.

☒ A numerical value for number of cells or percentage (with statistics) is provided.

Methodology

Sample preparation	Bacteria was grown to exponential growth and incubated with propidium iodide (PI) for 15 min in the dark.
Instrument	CytExpert Flow Cytometer (Beckman, USA)
Software	CytExpert 2.0 software (Beckman, USA)
Cell population abundance	1,000,000 cells for each sample.
Gating strategy	Negative control (untreated) and PI-positive cells were used to establish gates.

☒ Tick this box to confirm that a figure exemplifying the gating strategy is provided in the Supplementary Information.
